# Effect of Thomas Rotation on the Lorentz Transformation of Electromagnetic fields

**DOI:** 10.1038/s41598-020-62082-z

**Published:** 2020-03-26

**Authors:** Lakshya Malhotra, Robert Golub, Eva Kraegeloh, Nima Nouri, Bradley Plaster

**Affiliations:** 1 University of Kentucky, Department of Physics and Astronomy, Lexington, Kentucky 40508 USA; 2 North Carolina State University, Department of Physics, Raleigh, North Carolina 27695 USA; 3 University of Michigan, Department of Physics, Ann Arbor, Michigan 48109 USA; 4 Center for Medical Informatics, Yale School of Medicine, New Haven, Connecticut 06511 USA; 5 Present Address: Center for Medical Informatics, Yale School of Medicine, New Haven, Connecticut 06511 USA; 6 Present Address: Department of Pathology, Yale School of Medicine, New Haven, Connecticut 06511 USA

**Keywords:** Theoretical nuclear physics, Theoretical particle physics

## Abstract

A relativistic particle undergoing successive boosts which are non collinear will experience a rotation of its coordinate axes with respect to the boosted frame. This rotation of coordinate axes is caused by a relativistic phenomenon called Thomas Rotation. We assess the importance of Thomas rotation in the calculation of physical quantities like electromagnetic fields in the relativistic regime. We calculate the electromagnetic field tensor for general three dimensional successive boosts in the particle’s rest frame as well as the laboratory frame. We then compare the electromagnetic field tensors obtained by a direct boost $\overrightarrow{\beta }+\delta \overrightarrow{\beta }$ and successive boosts $\overrightarrow{\beta }$ and $\Delta \overrightarrow{\beta }$ and check their consistency with Thomas rotation. This framework might be important to situations such as the calculation of frequency shifts for relativistic spin-1/2 particles undergoing Larmor precession in electromagnetic fields with small field non-uniformities.

## Introduction

**Figure 1:**
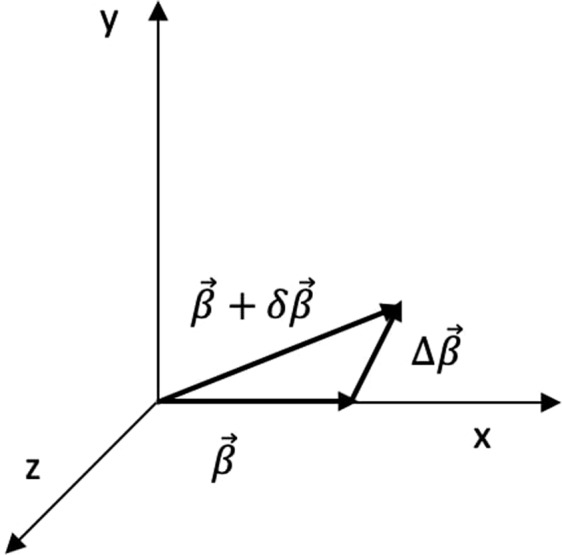
Schematic of the boosts. $\overrightarrow{\beta }+\delta \overrightarrow{\beta }$: Direct boost, $\overrightarrow{\beta }$: First successive boost, $\delta \overrightarrow{\beta }$: Second successive boost in lab frame, $\Delta \overrightarrow{\beta }$: Second successive boost with respect to the inertial frame with boost $\overrightarrow{\beta }$.

As pointed out by Thomas^1^, two successive non collinear Lorentz boosts are not equal to a direct boost but to a direct boost followed by a rotation of the coordinate axes. That is,

1 $$\begin{array}{lll}A(\overrightarrow{\beta }+\delta \overrightarrow{\beta }) & \ne  & A(\delta \overrightarrow{\beta })\cdot A(\overrightarrow{\beta })\\ A(\overrightarrow{\beta }+\delta \overrightarrow{\beta }) & = & {R}_{{\rm{tom}}}(\Delta \overrightarrow{\Omega })\cdot A(\Delta \overrightarrow{\beta })\cdot A(\overrightarrow{\beta })\end{array}$$

where $\left(\overrightarrow{\beta }+\delta \overrightarrow{\beta }\right)$ is the direct boost, $\overrightarrow{\beta }$ and $\delta \overrightarrow{\beta }$ are two successive boosts in the lab frame, $\Delta \overrightarrow{\beta }$ and $\Delta \overrightarrow{\Omega }$ (not shown) are, respectively, the successive boost and the angle of rotation with respect to the frame with boost $\overrightarrow{\beta }$ (Fig. 1). $A(\overrightarrow{\beta }+\delta \overrightarrow{\beta })$, $A(\overrightarrow{\beta }+\delta \overrightarrow{\beta })$, $A(\overrightarrow{\beta }),\,A(\delta \overrightarrow{\beta })$ and $A(\Delta \overrightarrow{\beta })$ are the usual boost matrices for the direct boost and the successive boosts respectively, and ${R}_{{\rm{tom}}}\left(\Delta \overrightarrow{\Omega }\right)$ is the rotation matrix^2,3^.

This rotation of the space coordinates under the application of successive Lorentz boosts is called Thomas rotation. This phenomenon occurs when a relativistic particle is undergoing accelerated motion. Now since we have to show the acceleration, we added an infinitesimal boost vector $\delta \overrightarrow{\beta }$ to the original boost $\overrightarrow{\beta }$.

In general, for boosts ${\overrightarrow{\beta }}_{1}$ and ${\overrightarrow{\beta }}_{2}$ which are parallel to each other or more specifically boosts corresponding to (1 + 1)-dimensional pure Lorentz transformations, the transformation matrix forms a group which satisfies the equation:

2 $$A\left({\overrightarrow{\beta }}_{1}\right)\cdot A\left({\overrightarrow{\beta }}_{2}\right)=A\left({\overrightarrow{\beta }}_{12}\right)$$

where *β*_12_ is the velocity composition of two boosts which is given by the equation:

3 $${\beta }_{12}=\frac{{\beta }_{1}+{\beta }_{2}}{1+{\beta }_{1}\cdot {\beta }_{2}}$$

But successive boosts which are non collinear, in general, result in Thomas rotation of the space coordinates or in other words, the boosted frames which are accelerating in the sense that their direction is changing will experience Thomas rotation. So the values of the physical quantities obtained by applying just Lorentz transformation are not correct in such cases.

This work is inspired by the ideas discussed in^4–11^ but in a slightly different manner. Ungar *et al*. defined three inertial reference frames Σ, ${\Sigma }^{{\prime} }$, and Σ^″^ in such a way that their corresponding axes are parallel to each other (Σ being the lab frame). It is assumed that the relative velocity of ${\Sigma }^{{\prime} }$ with respect to Σ and the relative velocity of Σ^″^ with respect to ${\Sigma }^{{\prime} }$ is known beforehand. The relativistic velocity composition law can then be used to calculate the velocity of Σ^″^ with respect to Σ. Usually the velocity of Σ^″^ with respect to ${\Sigma }^{{\prime} }$ is not known so the above mentioned approach cannot be used directly. To circumvent this issue, in this paper we present a calculation in which we calculated a matrix *A*_*T*_^3^ from $A\left(\overrightarrow{\beta }+\delta \overrightarrow{\beta }\right)$ and $A\left(-\overrightarrow{\beta }\right)$ which contains all the information about relativistic composition of velocities and Thomas rotation.

To our knowledge, the case of non-collinear boosts and its effects on the electromagnetic field tensor has not been discussed in the literature. The aim of this paper is to see how the electromagnetic field tensor transforms with the Lorentz transformations for general three-dimensional boosts and to show that the field tensor in the direct boosted frame $A\left(\overrightarrow{\beta }+\delta \overrightarrow{\beta }\right)$ and successive boosted frames $A(\overrightarrow{\beta })$ and $A\left(\delta \overrightarrow{\beta }\right)$ are consistent with Thomas rotation.

## Survey of some concepts of the Special Theory of Relativity

### Lorentz transformations

For two inertial reference frames Σ and ${\Sigma }^{{\prime} }$ which have a relative velocity of $\overrightarrow{v}$ in such a way that the coordinate axes of Σ are parallel to ${\Sigma }^{{\prime} }$ and ${\Sigma }^{{\prime} }$ is moving in the positive *x* direction as seen from Σ, the position 4-vector of ${\Sigma }^{{\prime} }$ is related to the position 4-vector of Σ by the standard Lorentz transformation equations^12^:

4 $$\begin{array}{ccc}{{x}^{{\rm{{\prime} }}}}_{0} & = & \gamma ({x}_{0}-\beta {x}_{1})\\ {{x}^{{\rm{{\prime} }}}}_{1} & = & \gamma ({x}_{1}-\beta {x}_{0})\\ {{x}^{{\rm{{\prime} }}}}_{2} & = & {x}_{2}\\ {{x}^{{\rm{{\prime} }}}}_{3} & = & {x}_{3}\end{array}$$

where

$$\begin{array}{ccl}{x}_{0} & = & ct,{x}_{1}=x,{x}_{2}=y,{x}_{3}=z;\\ \overrightarrow{\beta } & = & \frac{\overrightarrow{v}}{c},\beta =\left|\overrightarrow{\beta }\right|;\\ \gamma  & = & {(1-{\beta }^{2})}^{-1/2}:\ \ {\rm{Lorentz}}\ {\rm{factor}}\end{array}$$

The generalization of Eq. (4) for the relative velocity of ${\Sigma }^{{\prime} }$ in an arbitrary direction but with the coordinate axes of the two frames still parallel to each other is given by:

5 $$\begin{array}{ccc}{{x}^{{\rm{{\prime} }}}}_{0} & = & \gamma ({x}_{0}-\overrightarrow{\beta }\cdot \overrightarrow{x})\\ {\overrightarrow{x}}^{{\rm{{\prime} }}} & = & \overrightarrow{x}+\frac{(\gamma -1)}{{\beta }^{2}}(\overrightarrow{\beta }\cdot \overrightarrow{x})\overrightarrow{\beta }-\gamma \overrightarrow{\beta }{x}_{0}\end{array}$$

### Addition of velocities

Consider two inertial reference frames Σ and ${\Sigma }^{{\prime} }$ such that the relative velocity of ${\Sigma }^{{\prime} }$ with respect to Σ is $\overrightarrow{v}$. A particle is moving in ${\Sigma }^{{\prime} }$ such that its velocity with respect to ${\Sigma }^{{\prime} }$ is ${\overrightarrow{u}}^{{\prime} }$. The velocity of the particle with respect to Σ is then given by^13^:

6 $$\begin{array}{ccc}{u}_{\parallel } & = & \frac{{{u}^{{\rm{{\prime} }}}}_{\parallel }+v}{1+\frac{\overrightarrow{v}\cdot {\overrightarrow{u}}^{{\rm{{\prime} }}}}{{c}^{2}}}\\ {\overrightarrow{u}}_{\perp } & = & \frac{{\overrightarrow{u}}_{\perp }^{{\rm{{\prime} }}}}{{\gamma }_{v}\left(1,+,\frac{\overrightarrow{v}\cdot {\overrightarrow{u}}^{{\rm{{\prime} }}}}{{c}^{2}}\right)}\end{array}$$

where *u*_∥_ and ${\overrightarrow{u}}_{\perp }$ refer to the components of velocity parallel and perpendicular, respectively, to $\overrightarrow{v}$.

It can be shown that the Lorentz factor of $\overrightarrow{v}$, $\overrightarrow{u}$, and ${\overrightarrow{u}}^{{\prime} }$ are related to each other by

7 $${\gamma }_{u}={\gamma }_{v}{\gamma }_{{u}^{{\prime} }}\left(1+\frac{\overrightarrow{v}\cdot {\overrightarrow{u}}^{{\prime} }}{{c}^{2}}\right)$$

More generally, the velocity composition law for two arbitrary velocities can be written as^4–10,13^:

$$\overrightarrow{u}\oplus \overrightarrow{v}=\frac{\overrightarrow{u}+\overrightarrow{v}}{1+\frac{\overrightarrow{u}\cdot \overrightarrow{v}}{{c}^{2}}}+\frac{1}{{c}^{2}}\left(\frac{{\gamma }_{u}}{{\gamma }_{u}+1}\right)\frac{\overrightarrow{u}\times (\overrightarrow{u}\times \overrightarrow{v})}{1+\frac{\overrightarrow{u}\cdot \overrightarrow{v}}{{c}^{2}}}$$

with

8 $${\gamma }_{u\oplus v}={\gamma }_{u}{\gamma }_{v}\left(1+\frac{\overrightarrow{u}\cdot \overrightarrow{v}}{{c}^{2}}\right)$$

where symbol  ⊕  refers to the direct sum of the vector space of the velocity vectors.

### Matrix representation and boost matrix

For the rest of the paper, we will be using matrix methods to calculate Lorentz transformations as they are very convenient to use and are more explicit. All of the equations in Eqs. (4) and (5) can easily be obtained by using the boost matrices for Lorentz transformations. For example, for a boost along the *x* axis, the boost matrix can be written as^3^:

9 $$A=\left(\begin{array}{cccc}\gamma  & -\gamma \beta  & 0 & 0\\ -\gamma \beta  & \gamma  & 0 & 0\\ 0 & 0 & 1 & 0\\ 0 & 0 & 0 & 1\end{array}\right)$$

Hence,

10 $$(\begin{array}{c}c{t}^{{\rm{{\prime} }}}\\ {x}^{{\rm{{\prime} }}}\\ {y}^{{\rm{{\prime} }}}\\ {z}^{{\rm{{\prime} }}}\end{array})=(\begin{array}{cccc}\gamma  & -\gamma \beta  & 0 & 0\\ -\gamma \beta  & \gamma  & 0 & 0\\ 0 & 0 & 1 & 0\\ 0 & 0 & 0 & 1\end{array})(\begin{array}{c}ct\\ x\\ y\\ z\end{array})=(\begin{array}{c}\gamma (ct-\beta x)\\ \gamma (x-\beta ct)\\ y\\ z\end{array})$$

For an arbitrary boost, the general form of the boost matrix *A* takes a form in which the matrix elements can be written as:

11 $$\begin{array}{lll}{A}_{00} & = & \gamma \\ {A}_{0i} & = & {A}_{i0}=-\gamma {\beta }_{i}\\ {A}_{ij} & = & {A}_{ji}={\delta }_{ij}+(\gamma -1)\frac{{\beta }_{i}{\beta }_{j}}{{\beta }^{2}}\end{array}$$

where *δ*_*ij*_ is the kronecker delta. The general form of Eq. (9) can therefore be written as^2^:

12 $$A=\left(\begin{array}{cccc}\gamma  & -\gamma {\beta }_{x} & -\gamma {\beta }_{y} & -\gamma {\beta }_{z}\\ -\gamma {\beta }_{x} & 1+(\gamma -1)\frac{{\beta }_{x}^{2}}{{\beta }^{2}} & (\gamma -1)\frac{{\beta }_{x}{\beta }_{y}}{{\beta }^{2}} & (\gamma -1)\frac{{\beta }_{x}{\beta }_{z}}{{\beta }^{2}}\\ -\gamma {\beta }_{y} & (\gamma -1)\frac{{\beta }_{y}{\beta }_{x}}{{\beta }^{2}} & 1+(\gamma -1)\frac{{\beta }_{y}^{2}}{{\beta }^{2}} & (\gamma -1)\frac{{\beta }_{y}{\beta }_{z}}{{\beta }^{2}}\\ -\gamma {\beta }_{z} & (\gamma -1)\frac{{\beta }_{z}{\beta }_{x}}{{\beta }^{2}} & (\gamma -1)\frac{{\beta }_{z}{\beta }_{y}}{{\beta }^{2}} & 1+(\gamma -1)\frac{{\beta }_{z}^{2}}{{\beta }^{2}}\end{array}\right)$$

### Set-up

To start with, consider two arbitrary boosts $\overrightarrow{\beta }$ and $\delta \overrightarrow{\beta }$ in three dimensions:

13 $$\begin{array}{ccc}\overrightarrow{\beta } & = & {\beta }_{x}\hat{x}+{\beta }_{y}\hat{y}+{\beta }_{z}\hat{z}\\ \delta \overrightarrow{\beta } & = & \delta {\beta }_{x}\hat{x}+\delta {\beta }_{y}\hat{y}+\delta {\beta }_{z}\hat{z}\end{array}$$

In order to calculate the boost matrix for various boosts, we will apply a passive transformation which will rotate our lab frame (*xy*) coordinate axes in such a way that its *x*-axis is aligned with $\overrightarrow{\beta }$. This rotated frame will hereafter be called the longitudinal-transverse (*ℓt*) frame.

**Figure 2:**
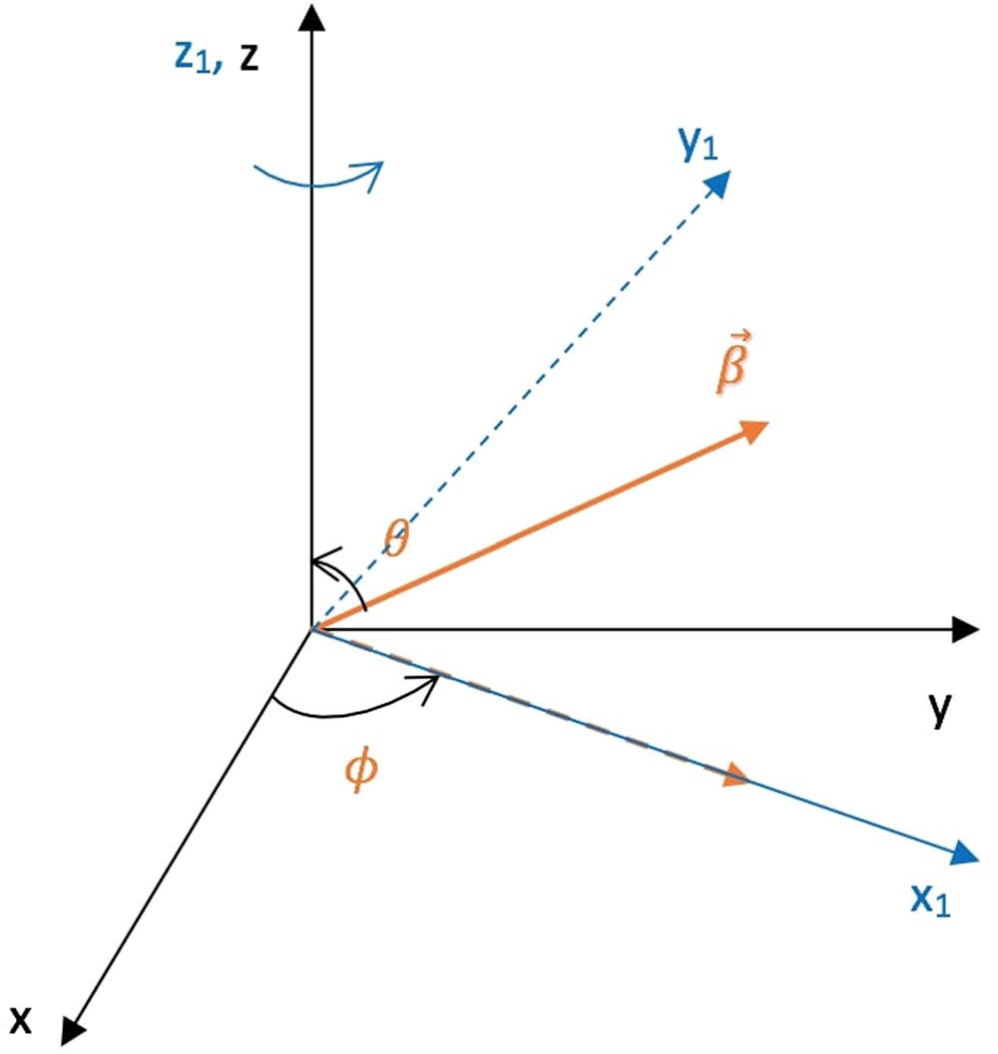
Rotation about *z* axis by an angle *ϕ*. The new *x*, *y* and *z* axes are called the *x*_1_, *y*_1_ and *z*_1_ axes respectively.

This whole transformation can be imagined as a product of two rotations: The first rotation is about the *z* axis by an angle *ϕ* which will align the *x* axis along the projection of the boost vector in the *xy* plane (Fig. 2). The rotation matrix associated with this rotation can be written as:

14 $${R}_{1}=\left(\begin{array}{cccc}1 & 0 & 0 & 0\\ 0 & \cos \phi  & \sin \phi  & 0\\ 0 & -\sin \phi  & \cos \phi  & 0\\ 0 & 0 & 0 & 1\end{array}\right)$$

**Figure 3:**
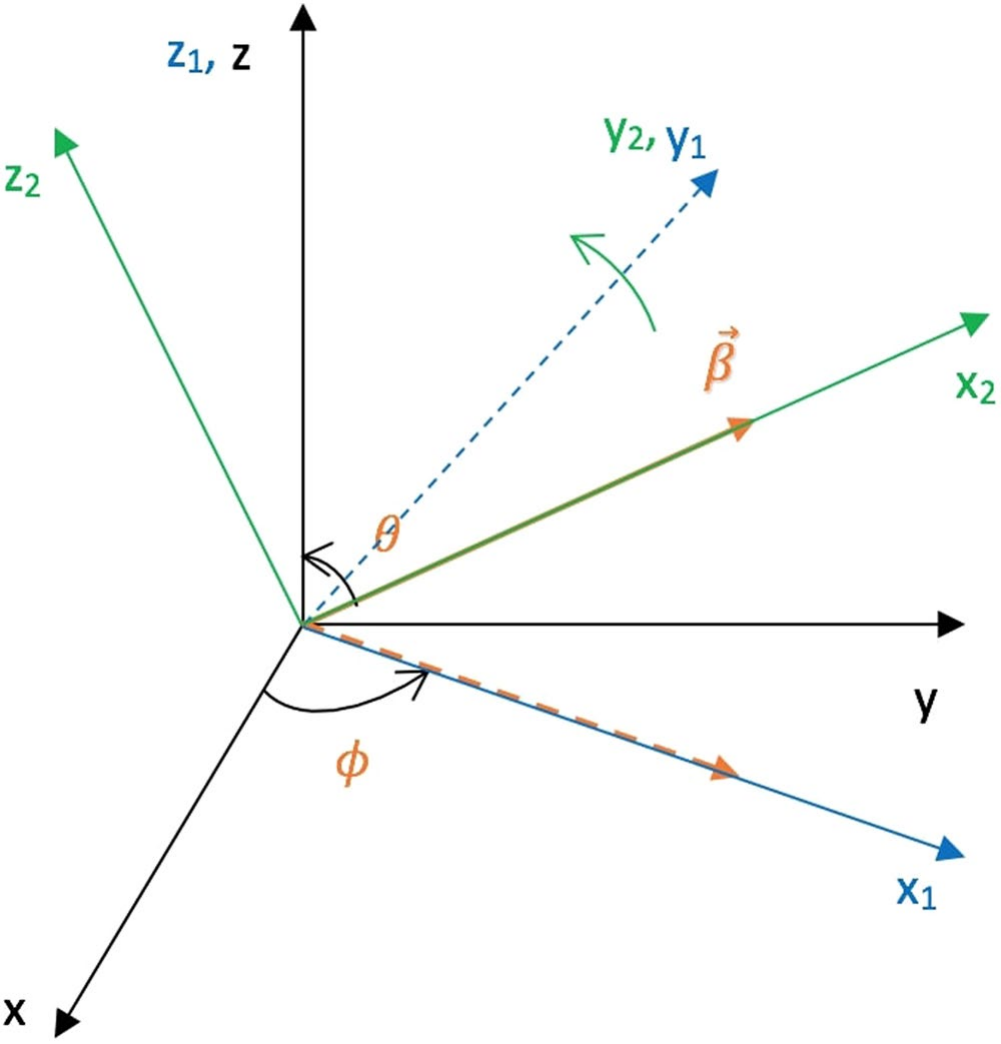
Second rotation about the *y*_1_ axis by an angle $\frac{\pi }{2}-\theta$. *x*, *y*, and *z* axes in this new frame are called the *x*_2_, *y*_2_ and *z*_2_ axes respectively.

The second rotation is about the *y*_1_ axis (Fig. 3) by an angle $\frac{\pi }{2}-\theta$. The effect of this rotation is that it aligns the *x*_1_ axis to the boost vector $\overrightarrow{\beta }$. For the second rotation, the rotation matrix can be written as:

15 $${R}_{2}=\left(\begin{array}{cccc}1 & 0 & 0 & 0\\ 0 & \sin \theta  & 0 & \cos \theta \\ 0 & 0 & 1 & 0\\ 0 & -\cos \theta  & 0 & \sin \theta \end{array}\right)$$

The overall effect of the two rotations can be combined in a single transformation matrix *R*:

16 $$R={R}_{2}\cdot {R}_{1}=\left(\begin{array}{cccc}1 & 0 & 0 & 0\\ 0 & \sin \theta \cos \phi  & \sin \theta \sin \phi  & \cos \theta \\ 0 & -\sin \phi  & \cos \phi  & 0\\ 0 & -\cos \theta \cos \phi  & -\cos \theta \sin \phi  & \sin \theta \end{array}\right)$$

**Figure 4:**
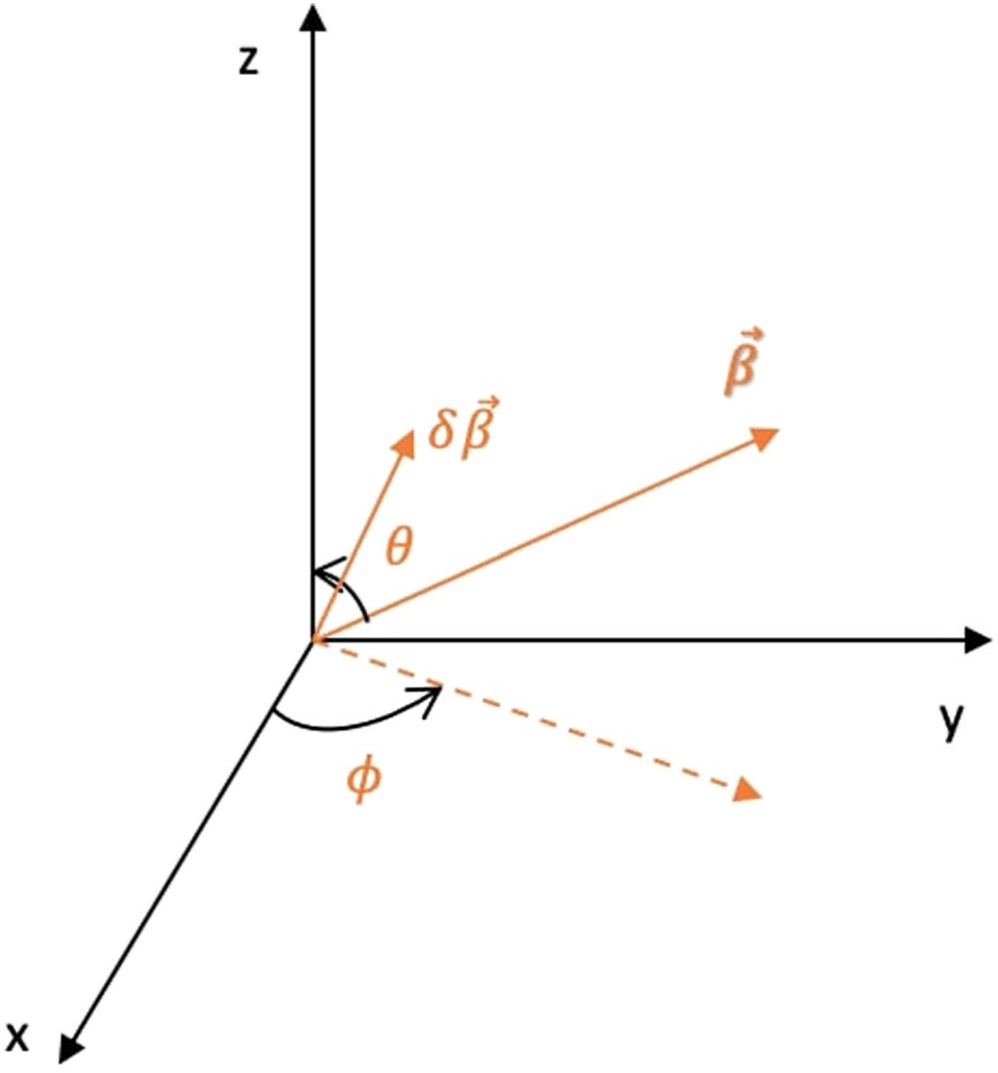
General boost in three-dimensions. Dotted line represents the projection of $\overrightarrow{\beta }$ on the *xy*-plane.

It is clear from Fig. 4 that if:

$$\overrightarrow{\beta }={\beta }_{x}\hat{x}+{\beta }_{y}\hat{y}+{\beta }_{z}\hat{z}$$

then

17 $$\cos \theta =\frac{{\beta }_{z}}{{\lambda }_{1}};\qquad \sin \theta =\frac{{\eta }_{1}}{{\lambda }_{1}};\qquad \cos \phi =\frac{{\beta }_{x}}{{\eta }_{1}};\qquad \sin \phi =\frac{{\beta }_{y}}{{\eta }_{1}}$$

where the parameters *λ*_1_ and *η*_1_ are defined in the Supplementary Information (Section 1).

Using Eq. (17), the matrix *R* can be written as:

18 $$R=\left(\begin{array}{cccc}1 & 0 & 0 & 0\\ 0 & \frac{{\beta }_{x}}{{\lambda }_{1}} & \frac{{\beta }_{y}}{{\lambda }_{1}} & \frac{{\beta }_{z}}{{\lambda }_{1}}\\ 0 & -\frac{{\beta }_{y}}{{\eta }_{1}} & \frac{{\beta }_{x}}{{\eta }_{1}} & 0\\ 0 & -\frac{{\beta }_{x}{\beta }_{z}}{{\eta }_{1}{\lambda }_{1}} & -\frac{{\beta }_{y}{\beta }_{z}}{{\eta }_{1}{\lambda }_{1}} & \frac{{\eta }_{1}}{{\lambda }_{1}}\end{array}\right)$$

As mentioned earlier, this rotation matrix will transform the lab frame coordinates of any 4-vector (*xy*-frame) to its coordinates in the rotating frame also called longitudinal-transverse frame (*ℓt*-frame):

19 $$\begin{array}{lll}{\overrightarrow{\beta }}^{\ell t} & = & R\cdot {\overrightarrow{\beta }}^{xy}\\ \left(\begin{array}{c}0\\ {\beta }_{x}^{\ell t}\\ {\beta }_{y}^{\ell t}\\ {\beta }_{z}^{\ell t}\end{array}\right) & = & R\cdot \left(\begin{array}{c}0\\ {\beta }_{x}^{xy}\\ {\beta }_{y}^{xy}\\ {\beta }_{z}^{xy}\end{array}\right)=\left(\begin{array}{c}0\\ {\lambda }_{1}\\ 0\\ 0\end{array}\right)\end{array}$$

where the superscripts *ℓt* and *xy* refer to the components in longitudinal-transverse frame and laboratory frame, respectively, and for the sake of simplicity in notation we assumed:

$${\beta }_{x}^{xy}={\beta }_{x},\qquad {\beta }_{y}^{xy}={\beta }_{y},\qquad {\beta }_{z}^{xy}={\beta }_{z};$$

Similarly, the infinitesimal boost in the longitudinal-transverse frame is of the form:

20 $$\begin{array}{lll}\delta {\overrightarrow{\beta }}^{\ell t} & = & R\cdot \delta {\overrightarrow{\beta }}^{xy}\\ \left(\begin{array}{c}0\\ \delta {\beta }_{x}^{\ell t}\\ \delta {\beta }_{y}^{\ell t}\\ \delta {\beta }_{z}^{\ell t}\end{array}\right) & = & R\cdot \left(\begin{array}{c}0\\ \delta {\beta }_{x}^{xy}\\ \delta {\beta }_{y}^{xy}\\ \delta {\beta }_{z}^{xy}\end{array}\right)=\left(\begin{array}{c}0\\ \frac{{\lambda }_{2}}{{\lambda }_{1}}\\ \frac{{\lambda }_{6}}{{\eta }_{1}}\\ \frac{{\lambda }_{5}}{{\eta }_{1}{\lambda }_{1}}\end{array}\right)\end{array}$$

where *λ*_1_, *λ*_2_, *λ*_5_, *λ*_6_ and *η*_1_ are defined in the Supplementary Information (Section 1).

To calculate the ${\gamma }_{(\overrightarrow{\beta }+\delta \overrightarrow{\beta })}$ in the *ℓt*-frame, we have:

$${(\overrightarrow{\beta }+\delta \overrightarrow{\beta })}^{\ell t}=\left({\lambda }_{1},+,\frac{{\lambda }_{2}}{{\lambda }_{1}}\right)\hat{x}+\frac{{\lambda }_{6}}{{\eta }_{1}}\hat{y}+\frac{{\lambda }_{5}}{{\eta }_{1}{\lambda }_{1}}\hat{z}$$

Keeping the terms linear in *δβ*, we get

$${\left|{\left(\overrightarrow{\beta }+\delta \overrightarrow{\beta }\right)}^{\ell t}\right|}^{2}\approx {\lambda }_{1}^{2}+2{\lambda }_{2}$$

Using the above equation we calculate:

$$\begin{array}{lll}{\gamma }_{(\overrightarrow{\beta }+\delta \overrightarrow{\beta })} & = & {\left(1-{\lambda }_{1}^{2}-2{\lambda }_{2}\right)}^{-\frac{1}{2}}\\  & \approx  & {\left(1-{\lambda }_{1}^{2}\right)}^{-\frac{1}{2}}\left[1+\frac{{\lambda }_{2}}{1-{\lambda }_{1}^{2}}\right]\end{array}$$

Hence, ${\gamma }_{(\overrightarrow{\beta }+\delta \overrightarrow{\beta })}$ can be written as:

$${\gamma }_{(\overrightarrow{\beta }+\delta \overrightarrow{\beta })}\approx \gamma (1+{\gamma }^{2}{\lambda }_{2})$$

where $\gamma =\frac{1}{\sqrt{1-{\lambda }_{1}^{2}}}$ is the Lorentz factor.

Using Eq. (19), the boost matrix for boost ${\overrightarrow{\beta }}^{\ell t}$ can be calculated:

21 $$A{\left(\overrightarrow{\beta }\right)}^{\ell t}=\left(\begin{array}{cccc}\gamma  & -\gamma {\lambda }_{1} & 0 & 0\\ -\gamma {\lambda }_{1} & \gamma  & 0 & 0\\ 0 & 0 & 1 & 0\\ 0 & 0 & 0 & 1\end{array}\right)$$

Similarly, using Eqs. (19) and (20), to the first order in *δβ*, the boost matrix for the direct boost $\overrightarrow{\beta }+\delta \overrightarrow{\beta }$ in the *ℓt*-frame can be written as:

22 $$A{(\overrightarrow{\beta }+\delta \overrightarrow{\beta })}^{\ell t}=\left(\begin{array}{cccc}\gamma +{\gamma }^{3}{\lambda }_{2} & -\frac{\gamma ({\lambda }_{1}^{2}+{\gamma }^{2}{\lambda }_{2})}{{\lambda }_{1}} & -\frac{\gamma {\lambda }_{6}}{{\eta }_{1}} & -\frac{\gamma {\lambda }_{5}}{{\eta }_{1}{\lambda }_{1}}\\ -\frac{\gamma ({\lambda }_{1}^{2}+{\gamma }^{2}{\lambda }_{2})}{{\lambda }_{1}} & \gamma +{\gamma }^{3}{\lambda }_{2} & \frac{(\gamma -1){\lambda }_{6}}{{\eta }_{1}{\lambda }_{1}} & \frac{(\gamma -1){\lambda }_{5}}{{\eta }_{1}{\lambda }_{1}^{2}}\\ -\frac{\gamma {\lambda }_{6}}{{\eta }_{1}} & \frac{(\gamma -1){\lambda }_{6}}{{\eta }_{1}{\lambda }_{1}} & 1 & 0\\ -\frac{\gamma {\lambda }_{5}}{{\eta }_{1}{\lambda }_{1}} & \frac{(\gamma -1){\lambda }_{5}}{{\eta }_{1}{\lambda }_{1}^{2}} & 0 & 1\end{array}\right)$$

## Transformations of the Electromagnetic Field Tensor

The main idea of this paper is to see how the electromagnetic fields transform relativistically when there is an accelerated motion. It can be further divided into transformations in the longitudinal-transverse and lab frame.

### Longitudinal-transverse ***ℓ****t*-frame

To see the effects on electromagnetic fields, we first need to bring the electromagnetic field tensor to the rotating *ℓt*-frame so that all the boosts and electromagnetic fields are in the same frame to start with. The electromagnetic field tensor *F*^*μν*^ in the lab frame is given by^14^:

23 $${F}^{\mu \nu }=\left(\begin{array}{cccc}0 & -{E}_{x} & -{E}_{y} & -{E}_{z}\\ {E}_{x} & 0 & -{B}_{z} & {B}_{y}\\ {E}_{y} & {B}_{z} & 0 & -{B}_{x}\\ {E}_{z} & -{B}_{y} & {B}_{x} & 0\end{array}\right)$$

For the rest of the paper we will write *F*^*μν*^ = *F*.

To get the field tensor in the *ℓt*-frame, we can apply the rotation matrix *R* on *F*^*μν*^:

24 $${F}^{\ell t}=R\cdot F\cdot {R}^{T}$$

where the superscript *T* refers to matrix transpose. After plugging in the values of *R* and *F* from Eqs. (18) and (23), we can write *F*^*ℓt*^ as:

$${F}^{\ell t}=\left(\begin{array}{cccc}0 & -\frac{{\kappa }_{1}}{{\lambda }_{1}} & -\frac{{\kappa }_{2}}{{\eta }_{1}} & -\frac{{\kappa }_{3}}{{\eta }_{1}{\lambda }_{1}}\\ \frac{{\kappa }_{1}}{{\lambda }_{1}} & 0 & -\frac{{\kappa }_{6}}{{\eta }_{1}{\lambda }_{1}} & \frac{{\kappa }_{5}}{{\eta }_{1}}\\ \frac{{\kappa }_{2}}{{\eta }_{1}} & \frac{{\kappa }_{6}}{{\eta }_{1}{\lambda }_{1}} & 0 & -\frac{{\kappa }_{4}}{{\lambda }_{1}}\\ \frac{{\kappa }_{3}}{{\eta }_{1}{\lambda }_{1}} & -\frac{{\kappa }_{5}}{{\eta }_{1}} & \frac{{\kappa }_{4}}{{\lambda }_{1}} & 0\end{array}\right)$$

where

$$\begin{array}{lll}{\kappa }_{1} & = & {\beta }_{x}{E}_{x}+{\beta }_{y}{E}_{y}+{\beta }_{z}{E}_{z}\\ {\kappa }_{2} & = & {\beta }_{x}{E}_{y}-{\beta }_{y}{E}_{x}\\ {\kappa }_{3} & = & {E}_{z}{\beta }_{x}^{2}-{\beta }_{x}{\beta }_{z}{E}_{x}+{\beta }_{y}\left({\beta }_{y}{E}_{z}-{\beta }_{z}{E}_{y}\right)\\ {\kappa }_{4} & = & {B}_{x}{\beta }_{x}+{B}_{y}{\beta }_{y}+{B}_{z}{\beta }_{z}\\ {\kappa }_{5} & = & {B}_{y}{\beta }_{x}-{B}_{x}{\beta }_{y}\\ {\kappa }_{6} & = & {B}_{z}{\eta }_{1}^{2}-\left({B}_{x}{\beta }_{x}+{B}_{y}{\beta }_{y}\right){\beta }_{z}\end{array}$$

To the electromagnetic field tensor obtained in Eq. (24), we will apply boost matrix for the first successive boost ${\overrightarrow{\beta }}^{\ell t}$ and the direct boost ${\left(\overrightarrow{\beta }+\delta \overrightarrow{\beta }\right)}^{\ell t}$ using the well-known equation^15^:

25 $${F}^{{\prime} }=A\cdot F\cdot {A}^{T}$$

where ${F}^{{\prime} }$ and *F* are the electromagnetic field tensors in the boosted frame and the lab frame (or any inertial frame) respectively and *A* is the boost matrix.

For boost ${\overrightarrow{\beta }}^{\ell t}$, the transformation of ${F}^{\ell t}$ can be calculated using Eqs. (21), (24) and (25):

$${({F}^{{\rm{{\prime} }}})}^{\ell t}=A({\overrightarrow{\beta }}^{\ell t})\cdot {F}^{\ell t}\cdot {(A({\overrightarrow{\beta }}^{\ell t}))}^{T}$$

The following table has the elements of ${({F}^{{\rm{{\prime} }}})}^{\ell t}$ after matrix multiplication: Electromagnetic fields in Table 1 are consistent with the standard field transformation equations^7,15^.

26 $$\begin{array}{lll}{\overrightarrow{E}}^{{\prime} } & = & \gamma \left(\overrightarrow{E}+\overrightarrow{\beta }\times \overrightarrow{B}\right)-\frac{{\gamma }^{2}}{\gamma +1}\ \overrightarrow{\beta }\left(\overrightarrow{\beta }\cdot \overrightarrow{E}\right)\\ {\overrightarrow{B}}^{{\prime} } & = & \gamma \left(\overrightarrow{B}-\overrightarrow{\beta }\times \overrightarrow{E}\right)-\frac{{\gamma }^{2}}{\gamma +1}\ \overrightarrow{\beta }\left(\overrightarrow{\beta }\cdot \overrightarrow{B}\right)\end{array}$$

Similarly, for the direct boost ${(\overrightarrow{\beta }+\delta \overrightarrow{\beta })}^{\ell t}$, the electromagnetic field tensor transformation is given by:

$${({F}^{{\rm{{\prime} }}{\rm{{\prime} }}})}^{\ell t}=A({(\overrightarrow{\beta }+\delta \overrightarrow{\beta })}^{\ell t})\cdot {F}^{\ell t}\cdot A{({(\overrightarrow{\beta }+\delta \overrightarrow{\beta })}^{\ell t})}^{T}$$

After simplification and keeping terms to linear order in *δβ*, (*F″*)^*ℓt*^ can be calculated and the detailed expressions of its elements are provided in the Supplementary Information (Section 2.1).

**Table 1:** Expressions for the indicated components of the electromagnetic field tensor after being transformed by the first boost ${\overrightarrow{\beta }}^{\ell t}$ in the longitudinal-transverse frame.

${({F{}^{{\rm{{\prime} }}}}^{10})}^{\ell t}$	${({E}_{x}^{{\prime} })}^{\ell t}$	$\frac{{\beta }_{x}{E}_{x}+{\beta }_{y}{E}_{y}+{\beta }_{z}{E}_{z}}{{\lambda }_{1}}$
${({F{}^{{\prime} }}^{20})}^{\ell t}$	${({E}_{y}^{{\prime} })}^{\ell t}$	$\frac{\gamma (-{B}_{z}{\eta }_{1}^{2}+{B}_{x}{\beta }_{x}{\beta }_{z}+{B}_{y}{\beta }_{y}{\beta }_{z}-{E}_{x}{\beta }_{y}+{\beta }_{x}{E}_{y})}{{\eta }_{1}}$
${({F{}^{{\prime} }}^{30})}^{\ell t}$	${({E}_{z}^{{\prime} })}^{\ell t}$	$\frac{\gamma ({B}_{y}{\beta }_{x}{\lambda }_{1}^{2}-{B}_{x}{\beta }_{y}{\lambda }_{1}^{2}+{\beta }_{x}^{2}{E}_{z}-{\beta }_{x}{E}_{x}{\beta }_{z}-{\beta }_{y}{E}_{y}{\beta }_{z}+{\beta }_{y}^{2}{E}_{z})}{{\eta }_{1}{\lambda }_{1}}$
${({F{}^{{\prime} }}^{32})}^{\ell t}$	${({B}_{x}^{{\prime} })}^{\ell t}$	$\frac{{B}_{x}{\beta }_{x}+{B}_{y}{\beta }_{y}+{B}_{z}{\beta }_{z}}{{\lambda }_{1}}$
${({F{}^{{\prime} }}^{13})}^{\ell t}$	${({B}_{y}^{{\prime} })}^{\ell t}$	$\frac{\gamma ({B}_{y}{\beta }_{x}-{B}_{x}{\beta }_{y}+{\beta }_{x}^{2}{E}_{z}-{\beta }_{x}{E}_{x}{\beta }_{z}-{\beta }_{y}{E}_{y}{\beta }_{z}+{\beta }_{y}^{2}{E}_{z})}{{\eta }_{1}}$
${({F{}^{{\prime} }}^{21})}^{\ell t}$	${({B}_{z}^{{\prime} })}^{\ell t}$	$\frac{\gamma ({B}_{z}{\eta }_{1}^{2}-{B}_{x}{\beta }_{x}{\beta }_{z}-{B}_{y}{\beta }_{y}{\beta }_{z}-{\beta }_{x}^{3}{E}_{y}+{\beta }_{x}^{2}{E}_{x}{\beta }_{y}-{\beta }_{x}{\beta }_{y}^{2}{E}_{y}+{E}_{x}{\beta }_{y}^{3}-{\beta }_{x}{E}_{y}{\beta }_{z}^{2}+{E}_{x}{\beta }_{y}{\beta }_{z}^{2})}{{\eta }_{1}{\lambda }_{1}}$

Because of the way we set up the problem, the electromagnetic field tensor described by the direct boost ${(\overrightarrow{\beta }+\delta \overrightarrow{\beta })}^{\ell t}$ already consists of rotations. To get the electromagnetic fields which do not have any rotations (pure Lorentz boost)^3^, we will use the successive boosts. As mentioned earlier in the Introduction, we will calculate a matrix *A*_*T*_:

27 $${A}_{T}=A\left(\overrightarrow{\beta }+\delta \overrightarrow{\beta }\right)\cdot A\left(-\overrightarrow{\beta }\right)$$

For the *ℓt*-frame, *A*_*T*_ looks like:

28 $${A}_{T}^{\ell t}=\left(\begin{array}{cccc}1 & \frac{-{\gamma }^{2}{\lambda }_{2}}{{\lambda }_{1}} & -\frac{\gamma {\lambda }_{6}}{{\eta }_{1}} & -\frac{\gamma {\lambda }_{5}}{{\eta }_{1}{\lambda }_{1}}\\ \frac{-{\gamma }^{2}{\lambda }_{2}}{{\lambda }_{1}} & 1 & \frac{(\gamma -1){\lambda }_{6}}{{\eta }_{1}{\lambda }_{1}} & \frac{(\gamma -1){\lambda }_{5}}{{\eta }_{1}{\lambda }_{1}^{2}}\\ -\frac{\gamma {\lambda }_{6}}{{\eta }_{1}} & -\frac{(\gamma -1){\lambda }_{6}}{{\eta }_{1}{\lambda }_{1}} & 1 & 0\\ -\frac{\gamma {\lambda }_{5}}{{\eta }_{1}{\lambda }_{1}} & -\frac{(\gamma -1){\lambda }_{5}}{{\eta }_{1}{\lambda }_{1}^{2}} & 0 & 1\end{array}\right)$$

The matrix *A*_*T*_ contains all the information regarding relativistic composition of velocities and Thomas rotation which can be seen if we write *A*_*T*_ as^3^:

29 $${A}_{T}^{\ell t}=A{(\Delta \overrightarrow{\beta })}^{\ell t}\cdot {R}_{{\rm{t}}{\rm{o}}{\rm{m}}}{(\Delta \overrightarrow{\Omega })}^{\ell t}=(I-\Delta {\overrightarrow{\beta }}^{\ell t}\cdot \overrightarrow{K})\cdot (I-\Delta {\overrightarrow{\Omega }}^{\ell t}\cdot \overrightarrow{S})$$

where: $\Delta {\overrightarrow{\beta }}^{\ell t}$ = successive boost with respect to frame with boost $\overrightarrow{\beta }$; $\Delta {\overrightarrow{\Omega }}^{\ell t}=\left[\left(\frac{\gamma -1}{{\beta }^{2}}\right){\overrightarrow{\beta }}^{\ell t}\times \delta {\overrightarrow{\beta }}^{\ell t}\right]$ is the angle of rotation associated with Thomas rotation.

It can be easily shown that if the boosts and rotations are infinitesimal then:

$$A\left(\Delta \overrightarrow{\beta }\right)\cdot {R}_{{\rm{tom}}}\left(\Delta \overrightarrow{\Omega }\right)={R}_{{\rm{tom}}}\left(\Delta \overrightarrow{\Omega }\right)\cdot A\left(\Delta \overrightarrow{\beta }\right)$$

Matrices $\overrightarrow{K}$ and $\overrightarrow{S}$ are the generators of Lorentz boosts and rotations respectively:

$$\begin{array}{lll}{K}_{1}=\left(\begin{array}{cccc}0 & 1 & 0 & 0\\ 1 & 0 & 0 & 0\\ 0 & 0 & 0 & 0\\ 0 & 0 & 0 & 0\end{array}\right), & {K}_{2}=\left(\begin{array}{cccc}0 & 0 & 1 & 0\\ 0 & 0 & 0 & 0\\ 1 & 0 & 0 & 0\\ 0 & 0 & 0 & 0\end{array}\right), & {K}_{3}=\left(\begin{array}{cccc}0 & 0 & 0 & 1\\ 0 & 0 & 0 & 0\\ 0 & 0 & 0 & 0\\ 1 & 0 & 0 & 0\end{array}\right)\\ {S}_{1}=\left(\begin{array}{cccc}0 & 0 & 0 & 0\\ 0 & 0 & 0 & 0\\ 0 & 0 & 0 & -1\\ 0 & 0 & 1 & 0\end{array}\right), & {S}_{2}=\left(\begin{array}{cccc}0 & 0 & 0 & 0\\ 0 & 0 & 0 & 1\\ 0 & 0 & 0 & 0\\ 0 & -1 & 0 & 0\end{array}\right), & {S}_{3}=\left(\begin{array}{cccc}0 & 0 & 0 & 0\\ 0 & 0 & -1 & 0\\ 0 & 1 & 0 & 0\\ 0 & 0 & 0 & 0\end{array}\right)\end{array}$$

Extracting the matrix form of $A(\Delta \overrightarrow{\beta }{)}^{\ell t}$ and ${R}_{{\rm{t}}{\rm{o}}{\rm{m}}}(\Delta \overrightarrow{\Omega }{)}^{\ell t}$ from *A*_*T*_, we get:

30 $$\begin{array}{lll}A{\left(\Delta \overrightarrow{\beta }\right)}^{\ell t} & = & \left(\begin{array}{cccc}1 & \frac{-{\gamma }^{2}{\lambda }_{2}}{{\lambda }_{1}} & -\frac{\gamma {\lambda }_{6}}{{\eta }_{1}} & -\frac{\gamma {\lambda }_{5}}{{\eta }_{1}{\lambda }_{1}}\\ \frac{-{\gamma }^{2}{\lambda }_{2}}{{\lambda }_{1}} & 1 & 0 & 0\\ -\frac{\gamma {\lambda }_{6}}{{\eta }_{1}} & 0 & 1 & 0\\ -\frac{\gamma {\lambda }_{5}}{{\eta }_{1}{\lambda }_{1}} & 0 & 0 & 1\end{array}\right)\\ {R}_{{\rm{tom}}}{\left(\Delta \overrightarrow{\Omega }\right)}^{\ell t} & = & \left(\begin{array}{cccc}1 & 0 & 0 & 0\\ 0 & 1 & \frac{(\gamma -1){\lambda }_{6}}{{\eta }_{1}{\lambda }_{1}} & \frac{(\gamma -1){\lambda }_{5}}{{\eta }_{1}{\lambda }_{1}^{2}}\\ 0 & -\frac{(\gamma -1){\lambda }_{6}}{{\eta }_{1}{\lambda }_{1}} & 1 & 0\\ 0 & -\frac{(\gamma -1){\lambda }_{5}}{{\eta }_{1}{\lambda }_{1}^{2}} & 0 & 1\end{array}\right)\end{array}$$

In order to find the electromagnetic fields due to pure Lorentz boosts, we calculate the electromagnetic field tensor due to the successive boosts ${\overrightarrow{\beta }}^{\ell t}$ and $\Delta {\overrightarrow{\beta }}^{\ell t}$:

31 $$\begin{array}{lll}{({F}^{\prime\prime\prime })}^{\ell t} & = & A{\left(\Delta \overrightarrow{\beta }\right)}^{\ell t}\cdot A{\left(\overrightarrow{\beta }\right)}^{\ell t}\cdot {F}^{\ell t}\cdot {\left(A{\left(\overrightarrow{\beta }\right)}^{\ell t}\right)}^{T}\cdot {\left(A{\left(\Delta \overrightarrow{\beta }\right)}^{\ell t}\right)}^{T}\\  & = & A{\left(\Delta \overrightarrow{\beta }\right)}^{\ell t}\cdot {({F}^{{\prime} })}^{\ell t}\cdot {\left(A{\left(\Delta \overrightarrow{\beta }\right)}^{\ell t}\right)}^{T}\end{array}$$

After simplification and keeping the terms which are linear in *δβ*, we get the matrix ${({F}^{\prime\prime\prime })}^{\ell t}$ whose elements are provided in the Supplementary Information (Section 2.2).

It should be noted that since ${({F}^{\prime\prime\prime })}^{\ell t}$ and (*F*″)^*ℓt*^ are different from each other by just a rotation, so ${({F}^{\prime\prime\prime })}^{\ell t}$ can be obtained by operating an inverse Thomas rotation on (*F″* )^*ℓt*^. In fact, we used this as a check for verifying if the expressions of electromagnetic fields calculated using Eq. (32) are correct.

32 $${({F}^{\prime\prime\prime })}^{\ell t}={R}_{{\rm{tom}}}{\left(-\Delta \overrightarrow{\Omega }\right)}^{\ell t}\cdot {({F}^{{\prime\prime} })}^{\ell t}\cdot {\left({R}_{{\rm{tom}}}{\left(-\Delta \overrightarrow{\Omega }\right)}^{\ell t}\right)}^{T}$$

### Laboratory *xy*-frame

After getting the expressions of electromagnetic fields in the *ℓt*-frame obtained by different boosts, we now calculate the electromagnetic fields by the same boosts with respect to the lab frame. The overall approach stays the same but all the boost matrices are needed to be transformed in the *xy*-frame before being used to calculate the electromagnetic field tensor. Another way of calculating the electromagnetic field tensor is to directly transform the field tensors obtained in *ℓt*-frame.

In order to calculate the electromagnetic field tensor for various boosts in the lab *xy*-frame, we will just use the field tensor *F* as defined in Eq. (23). Since *R* is the rotation matrix for passive coordinate transformations (18), we have:

33 $$R\cdot {R}^{T}={R}^{T}\cdot R=I$$

therefore we can write the electromagnetic tensors and boost matrices in the lab *xy*-frame as:

34 $$\begin{array}{lll}{F}^{xy} & = & {R}^{T}\cdot {F}^{\ell t}\cdot R\\ {A}^{xy} & = & {R}^{T}\cdot {A}^{\ell t}\cdot R\end{array}$$

After matrix multiplication, $A{\left(\overrightarrow{\beta }\right)}^{xy}$ can be written as:

35 $$A{\left(\overrightarrow{\beta }\right)}^{xy}=\left(\begin{array}{cccc}\gamma  & -\gamma {\beta }_{x} & -\gamma {\beta }_{y} & -\gamma {\beta }_{z}\\ -\gamma {\beta }_{x} & \frac{\gamma {\beta }_{x}^{2}+{\beta }_{y}^{2}+{\beta }_{z}^{2}}{{\lambda }_{1}^{2}} & \frac{(\gamma -1){\beta }_{x}{\beta }_{y}}{{\lambda }_{1}^{2}} & \frac{(\gamma -1){\beta }_{x}{\beta }_{z}}{{\lambda }_{1}^{2}}\\ -\gamma {\beta }_{y} & \frac{(\gamma -1){\beta }_{x}{\beta }_{y}}{{\lambda }_{1}^{2}} & \frac{{\beta }_{x}^{2}+\gamma {\beta }_{y}^{2}+{\beta }_{z}^{2}}{{\lambda }_{1}^{2}} & \frac{(\gamma -1){\beta }_{y}{\beta }_{z}}{{\lambda }_{1}^{2}}\\ -\gamma {\beta }_{z} & \frac{(\gamma -1){\beta }_{x}{\beta }_{z}}{{\lambda }_{1}^{2}} & \frac{(\gamma -1){\beta }_{y}{\beta }_{z}}{{\lambda }_{1}^{2}} & \frac{{\beta }_{x}^{2}+{\beta }_{y}^{2}+\gamma {\beta }_{z}^{2}}{{\lambda }_{1}^{2}}\end{array}\right)$$

which is in agreement with Eq. (12) if we substitute in

$$\overrightarrow{\beta }={\overrightarrow{\beta }}^{xy}={\beta }_{x}\hat{x}+{\beta }_{y}\hat{y}+{\beta }_{z}\hat{z}$$

Using Eqs. (25) and (34) we can calculate the electromagnetic field tensor ${({F}^{{\prime} })}^{xy}$ which corresponds to the boost ${\overrightarrow{\beta }}^{xy}$:

Again, the components of the electromagnetic field tensor in Table 2 can be verified from the standard field transformations as shown in Eq. (26).

**Table 2:** Expressions for the indicated components of the electromagnetic field tensor after being transformed by the first boost ${\overrightarrow{\beta }}^{xy}$ in the laboratory frame.

${({F{}^{{\prime} }}^{10})}^{xy}$	${({E}_{x}^{{\prime} })}^{xy}$	$\gamma {E}_{x}+\gamma \left({B}_{z}{\beta }_{y}-{B}_{y}{\beta }_{z}\right)-\frac{{\gamma }^{2}{\beta }_{x}\left({\beta }_{x}{E}_{x}+{\beta }_{y}{E}_{y}+{\beta }_{z}{E}_{z}\right)}{\gamma +1}$
${({F{}^{{\prime} }}^{20})}^{xy}$	${({E}_{y}^{{\prime} })}^{xy}$	$\gamma {E}_{y}+\gamma \left({B}_{x}{\beta }_{z}-{B}_{z}{\beta }_{x}\right)-\frac{{\gamma }^{2}{\beta }_{y}\left({\beta }_{x}{E}_{x}+{\beta }_{y}{E}_{y}+{\beta }_{z}{E}_{z}\right)}{\gamma +1}$
${({F{}^{{\prime} }}^{30})}^{xy}$	${({E}_{z}^{{\prime} })}^{xy}$	$\gamma {E}_{z}+\gamma \left({B}_{y}{\beta }_{x}-{B}_{x}{\beta }_{y}\right)-\frac{{\gamma }^{2}{\beta }_{z}\left({\beta }_{x}{E}_{x}+{\beta }_{y}{E}_{y}+{\beta }_{z}{E}_{z}\right)}{\gamma +1}$
$({F{}^{{\prime} }}^{32})xy$	${({B}_{x}^{{\prime} })}^{xy}$	$\gamma {B}_{x}+\gamma \left({E}_{y}{\beta }_{z}-{\beta }_{y}{E}_{z}\right)-\frac{{\gamma }^{2}{\beta }_{x}\left({B}_{x}{\beta }_{x}+{B}_{y}{\beta }_{y}+{B}_{z}{\beta }_{z}\right)}{\gamma +1}$
${({F{}^{{\prime} }}^{13})}^{xy}$	${({B}_{y}^{{\prime} })}^{xy}$	$\gamma {B}_{y}+\gamma \left({E}_{z}{\beta }_{x}-{\beta }_{z}{E}_{x}\right)-\frac{{\gamma }^{2}{\beta }_{x}\left({B}_{x}{\beta }_{x}+{B}_{y}{\beta }_{y}+{B}_{z}{\beta }_{z}\right)}{\gamma +1}$
${({F{}^{{\prime} }}^{21})}^{xy}$	${({B}_{z}^{{\prime} })}^{xy}$	$\gamma {B}_{z}+\gamma \left({E}_{x}{\beta }_{y}-{\beta }_{x}{E}_{y}\right)-\frac{{\gamma }^{2}{\beta }_{x}\left({B}_{x}{\beta }_{x}+{B}_{y}{\beta }_{y}+{B}_{z}{\beta }_{z}\right)}{\gamma +1}$

Similarly, for the direct boost ${(\overrightarrow{\beta }+\delta \overrightarrow{\beta })}^{xy}$, we can use Eq. (34) to calculate the boost matrix. The detailed expression of $A{(\overrightarrow{\beta }+\delta \overrightarrow{\beta })}^{xy}$ is too long to write here but it shares the same features as^3^ which can be seen if we let *δβ*_*z*_ = *β*_*y*_ = *β*_*z*_ = 0:

36 $$A{\left(\overrightarrow{\beta }+\delta \overrightarrow{\beta }\right)}^{xy}=\left(\begin{array}{cccc}\gamma +{\gamma }^{3}{\beta }_{x}\delta {\beta }_{x} & -(\gamma {\beta }_{x}+{\gamma }^{3}\delta {\beta }_{x}) & -\gamma \delta {\beta }_{y} & 0\\ -(\gamma {\beta }_{x}+{\gamma }^{3}\delta {\beta }_{x}) & \gamma +{\gamma }^{3}{\beta }_{x}\delta {\beta }_{x} & \left(\frac{\gamma -1}{{\beta }_{x}^{2}}\right){\beta }_{x}\delta {\beta }_{y} & 0\\ -\gamma \delta {\beta }_{y} & \left(\frac{\gamma -1}{{\beta }_{x}^{2}}\right){\beta }_{x}\delta {\beta }_{y} & 0 & 1\\ 0 & 0 & 0 & 1\end{array}\right)$$

The above matrix is identical in form to the one shown in^3^. After calculating the boost matrix Eq. (36), we can again use Eq. (25) to calculate the electromagnetic field tensor in the direct boosted frame with respect to the laboratory frame whose detailed expressions are provided in the Supplementary Information (Section 3.1).

In order to calculate electromagnetic fields in the inertial frames which are boosted upon by pure Lorentz boosts (no rotation), we use successive boosts $\overrightarrow{\beta }$ and $\Delta \overrightarrow{\beta }$. For that we have to calculate the expression of ${A}_{T}^{xy}$ first as done in Eq. (27) which is:

37 $${A}_{T}^{xy}=\left(\begin{array}{cccc}1 & -\frac{1}{{\lambda }_{1}^{2}}(\gamma {\lambda }_{3}+{\gamma }^{2}{\beta }_{x}{\lambda }_{2}) & -\frac{1}{{\lambda }_{1}^{2}}(\gamma {\lambda }_{4}+{\gamma }^{2}{\beta }_{y}{\lambda }_{2}) & -\frac{1}{{\lambda }_{1}^{2}}(\gamma {\lambda }_{5}+{\gamma }^{2}{\beta }_{z}{\lambda }_{2})\\ -\frac{1}{{\lambda }_{1}^{2}}(\gamma {\lambda }_{3}+{\gamma }^{2}{\beta }_{x}{\lambda }_{2}) & 1 & \frac{(\gamma -1){\lambda }_{6}}{{\lambda }_{1}^{2}} & -\frac{(\gamma -1){\lambda }_{8}}{{\lambda }_{1}^{2}}\\ -\frac{1}{{\lambda }_{1}^{2}}(\gamma {\lambda }_{4}+{\gamma }^{2}{\beta }_{y}{\lambda }_{2}) & -\frac{(\gamma -1){\lambda }_{6}}{{\lambda }_{1}^{2}} & 1 & \frac{(\gamma -1){\lambda }_{7}}{{\lambda }_{1}^{2}}\\ -\frac{1}{{\lambda }_{1}^{2}}(\gamma {\lambda }_{5}+{\gamma }^{2}{\beta }_{z}{\lambda }_{2}) & \frac{(\gamma -1){\lambda }_{8}}{{\lambda }_{1}^{2}} & -\frac{(\gamma -1){\lambda }_{7}}{{\lambda }_{1}^{2}} & 1\end{array}\right)$$

As we know from Eq. (29), $A{(\Delta \overrightarrow{\beta })}^{xy}$ and ${R}_{{\rm{tom}}}{(\Delta \overrightarrow{\Omega })}^{xy}$ can be extracted from ${A}_{T}^{xy}$ which can be written as:

38 $$\begin{array}{lll}A{\left(\Delta \overrightarrow{\beta }\right)}^{xy} & = & \left(\begin{array}{cccc}1 & -\frac{1}{{\lambda }_{1}^{2}}(\gamma {\lambda }_{3}+{\gamma }^{2}{\beta }_{x}{\lambda }_{2}) & -\frac{1}{{\lambda }_{1}^{2}}(\gamma {\lambda }_{4}+{\gamma }^{2}{\beta }_{y}{\lambda }_{2}) & -\frac{1}{{\lambda }_{1}^{2}}(\gamma {\lambda }_{5}+{\gamma }^{2}{\beta }_{z}{\lambda }_{2})\\ -\frac{1}{{\lambda }_{1}^{2}}(\gamma {\lambda }_{3}+{\gamma }^{2}{\beta }_{x}{\lambda }_{2}) & 1 & 0 & 0\\ -\frac{1}{{\lambda }_{1}^{2}}(\gamma {\lambda }_{4}+{\gamma }^{2}{\beta }_{y}{\lambda }_{2}) & 0 & 1 & 0\\ -\frac{1}{{\lambda }_{1}^{2}}(\gamma {\lambda }_{5}+{\gamma }^{2}{\beta }_{z}{\lambda }_{2}) & 0 & 0 & 1\end{array}\right)\\ {R}_{{\rm{tom}}}{\left(\Delta \overrightarrow{\Omega }\right)}^{xy} & = & \left(\begin{array}{cccc}1 & 0 & 0 & 0\\ 0 & 1 & \frac{(\gamma -1){\lambda }_{6}}{{\lambda }_{1}^{2}} & -\frac{(\gamma -1){\lambda }_{8}}{{\lambda }_{1}^{2}}\\ 0 & -\frac{(\gamma -1){\lambda }_{6}}{{\lambda }_{1}^{2}} & 1 & \frac{(\gamma -1){\lambda }_{7}}{{\lambda }_{1}^{2}}\\ 0 & \frac{(\gamma -1){\lambda }_{8}}{{\lambda }_{1}^{2}} & -\frac{(\gamma -1){\lambda }_{7}}{{\lambda }_{1}^{2}} & 1\end{array}\right)\end{array}$$

where *λ*_*i*_, (*i* = 1, 2, … , 8) and *η*_1_ are defined in the Supplementary Information (Section 1).

Using Eq. (38) we can now calculate electromagnetic fields due to pure Lorentz boosts whose detailed expressions are provided in the Supplementary Information (Section 3.2).

39 $$\begin{array}{lll}{({F}^{\prime\prime\prime })}^{xy} & = & A{\left(\Delta \overrightarrow{\beta }\right)}^{xy}\cdot A{\left(\overrightarrow{\beta }\right)}^{xy}\cdot {F}^{xy}\cdot {\left(A{(\overrightarrow{\beta })}^{xy}\right)}^{T}\cdot {\left(A{\left(\Delta \overrightarrow{\beta }\right)}^{xy}\right)}^{T}\\  & = & A{\left(\Delta \overrightarrow{\beta }\right)}^{xy}\cdot {({F}^{{\prime} })}^{xy}\cdot {\left(A{\left(\Delta \overrightarrow{\beta }\right)}^{xy}\right)}^{T}\end{array}$$

## Validation of Results

All the framework that we have constructed can be verified by two ways:

1. Verifying the form of boost matrices and electromagnetic field tensor for some special cases as discussed here^3,13^.
2. Applying this whole formalism on a 4-vector like position.

For the first approach, in order to see the identical nature of results we will assume the special case of *β*_*y*_ = *β*_*z*_ = *δβ*_*z*_ = 0. Applying this assumption on Eqs. (21) and (22) will give us:

40 $$\begin{array}{lll}A{\left(\overrightarrow{\beta }\right)}^{\ell t} & = & \left(\begin{array}{cccc}\gamma  & -\gamma {\beta }_{x} & 0 & 0\\ -\gamma {\beta }_{x} & \gamma  & 0 & 0\\ 0 & 0 & 1 & 0\\ 0 & 0 & 0 & 1\end{array}\right)\\ A{\left(\overrightarrow{\beta }+\delta \overrightarrow{\beta }\right)}^{\ell t} & = & \left(\begin{array}{cccc}\gamma +{\gamma }^{3}{\beta }_{x}\delta {\beta }_{x} & -(\gamma {\beta }_{x}+{\gamma }^{3}\delta {\beta }_{x}) & -\gamma \delta {\beta }_{y} & 0\\ -(\gamma {\beta }_{x}+{\gamma }^{3}\delta {\beta }_{x}) & \gamma +{\gamma }^{3}{\beta }_{x}\delta {\beta }_{x} & \left(\frac{\gamma -1}{{\beta }_{x}}\right)\delta {\beta }_{y} & 0\\ -\gamma \delta {\beta }_{y} & \left(\frac{\gamma -1}{{\beta }_{x}}\right)\delta {\beta }_{y} & 1 & 0\\ 0 & 0 & 0 & 1\end{array}\right)\end{array}$$

Similarly, ${A}_{T}^{\ell t}$ can be reduced to a familiar result^3^:

41 $${A}_{T}^{\ell t}=\left(\begin{array}{cccc}1 & -{\gamma }^{2}\delta {\beta }_{x} & -\gamma \delta {\beta }_{y} & 0\\ -{\gamma }^{2}\delta {\beta }_{x} & 1 & \frac{(\gamma -1)\delta {\beta }_{y}}{{\beta }_{x}} & 0\\ -\gamma \delta {\beta }_{y} & -\frac{(\gamma -1)\delta {\beta }_{y}}{{\beta }_{x}} & 1 & 0\\ 0 & 0 & 0 & 1\end{array}\right)$$

In the lab *xy*-frame, we get the exact same results as Eqs. (40) and (41) for the above mentioned special case. This makes perfect sense since letting *β*_*y*_ = *β*_*z*_ = *δβ*_*z*_ = 0 would just make the original passive coordinate transformations redundant and both the *ℓt*- and *xy*- frames will be identical.

To see if the matrix for Thomas rotation ${R}_{{\rm{tom}}}(\Delta \overrightarrow{\Omega })$ is correct we can directly calculate it from its definition:

42 $${R}_{{\rm{tom}}}\left(\Delta \overrightarrow{\Omega }\right)=\left(I-\Delta \overrightarrow{\Omega }\cdot \overrightarrow{S}\right)$$

where

$$\Delta \overrightarrow{\Omega }=\left[\left(\frac{\gamma -1}{{\beta }^{2}}\right)\overrightarrow{\beta }\times \delta \overrightarrow{\beta }\right]$$

The Thomas rotation matrix calculated from the Eq. (42) using the corresponding representations of the boost vectors in *ℓt*/*xy* -frames matches with Eqs. (30) and (38).

For the verification of Electromagnetic Field Tensors, we can calculate them in different ways. As an example, we calculated ${({F}^{\prime\prime\prime })}^{xy}$ using:

$$\begin{array}{lll}{({F}^{\prime\prime\prime })}^{xy} & = & A{\left(\Delta \overrightarrow{\beta }\right)}^{xy}\cdot A{\left(\overrightarrow{\beta }\right)}^{xy}\cdot {F}^{xy}\cdot {\left(A{\left(\overrightarrow{\beta }\right)}^{xy}\right)}^{T}\cdot {\left(A{\left(\Delta \overrightarrow{\beta }\right)}^{xy}\right)}^{T}\\  & = & {R}_{{\rm{tom}}}{\left(-\Delta \overrightarrow{\Omega }\right)}^{xy}\cdot {({F}^{{\prime\prime} })}^{xy}\cdot {\left({R}_{{\rm{tom}}}{\left(-\Delta \overrightarrow{\Omega }\right)}^{xy}\right)}^{T}\\  & = & {R}^{T}\cdot {({F}^{\prime\prime\prime })}^{\ell t}\cdot R\end{array}$$

All three equations yielded same results. Similar verification also holds for other electromagnetic field tensors involved.

Our second approach for verification is based on Ungar *et al*.^4,12,15^ in which we apply direct boost $(\overrightarrow{\beta }+\delta \overrightarrow{\beta })$ and successive boosts $\overrightarrow{\beta }$ and $\Delta \overrightarrow{\beta }$ to a position 4-vector. We can check if the results are consistent and share the same overall features as the electromagnetic field tensor. To see that we start with a general position 4-vector in the lab frame and for simplicity, we ignore the time component in the position 4-vector:

43 $${(r)}^{xy}=\left(\begin{array}{c}0\\ x\\ y\\ z\\ \end{array}\right)$$

Transforming it in the *ℓt*- frame using the rotation matrix *R* Eq. (18), we get:

44 $${(r)}^{\ell t}=R\cdot {r}^{xy}=\left(\begin{array}{c}0\\ \frac{x{\beta }_{x}+y{\beta }_{y}+z{\beta }_{z}}{{\lambda }_{1}}\\ \frac{y{\beta }_{x}-x{\beta }_{y}}{{\eta }_{1}}\\ \frac{z{\beta }_{x}^{2}-x{\beta }_{z}{\beta }_{x}+{\beta }_{y}\left(z{\beta }_{y}-y{\beta }_{z}\right)}{{\eta }_{1}{\lambda }_{1}}\end{array}\right)$$

We can calculate the expression of (*r*)^*ℓt*^ transformed by the first successive boost ${\overrightarrow{\beta }}^{\ell t}$ using Eq. (21) in the same way we calculated the electromagnetic field tensor *F*^*μν*^:

45 $${({r}^{{\prime} })}^{\ell t}=A{\left(\overrightarrow{\beta }\right)}^{\ell t}\cdot {(r)}^{\ell t}=\left(\begin{array}{c}-\gamma \left(x{\beta }_{x}+y{\beta }_{y}+z{\beta }_{z}\right)\\ \frac{\gamma \left(x{\beta }_{x}+y{\beta }_{y}+z{\beta }_{z}\right)}{{\lambda }_{1}}\\ \frac{y{\beta }_{x}-x{\beta }_{y}}{{\eta }_{1}}\\ \frac{z{\beta }_{x}^{2}-x{\beta }_{z}{\beta }_{x}+{\beta }_{y}\left(z{\beta }_{y}-y{\beta }_{z}\right)}{{\eta }_{1}{\lambda }_{1}}\end{array}\right)$$

which is nothing but the standard Lorentz transformation of coordinates. Similarly, for the direct boost ${(\overrightarrow{\beta }+\delta \overrightarrow{\beta })}^{\ell t}$ and successive boosts ${\overrightarrow{\beta }}^{\ell t}$ and $\Delta {\overrightarrow{\beta }}^{\ell t}$, after letting *β*_*z*_ = *δβ*_*z*_ = 0 for simplicity, we have:

46 $$\begin{array}{lll}{({r}^{{\prime\prime} })}^{\ell t} & = & A{(\overrightarrow{\beta }+\delta \overrightarrow{\beta })}^{\ell t}\cdot {(r)}^{\ell t}\\  & = & \left(\begin{array}{c}-\frac{\gamma \left(x{\beta }_{x}^{3}+\left(x\delta {\beta }_{x}{\gamma }^{2}+y{\beta }_{y}+y\delta {\beta }_{y}\right){\beta }_{x}^{2}+{\beta }_{y}\left(x{\beta }_{y}+\left({\gamma }^{2}-1\right)\left(y\delta {\beta }_{x}+x\delta {\beta }_{y}\right)\right){\beta }_{x}+{\beta }_{y}^{2}\left(y\delta {\beta }_{y}{\gamma }^{2}+y{\beta }_{y}+x\delta {\beta }_{x}\right)\right)}{{\eta }_{1}^{2}}\\ \frac{(\gamma -1)\left(y{\beta }_{x}-x{\beta }_{y}\right)\left({\beta }_{x}\delta {\beta }_{y}-{\beta }_{y}\delta {\beta }_{x}\right)+\left(x{\beta }_{x}+y{\beta }_{y}\right){\eta }_{1}^{2}\left(\left({\beta }_{x}\delta {\beta }_{x}+{\beta }_{y}\delta {\beta }_{y}\right){\gamma }^{3}+\gamma \right)}{{\eta }_{1}^{3}}\\ \,\frac{y{\beta }_{x}^{3}-x\left({\beta }_{y}-(\gamma -1)\delta {\beta }_{y}\right){\beta }_{x}^{2}+{\beta }_{y}\left(y{\beta }_{y}-(\gamma -1)\left(x\delta {\beta }_{x}-y\delta {\beta }_{y}\right)\right){\beta }_{x}-{\beta }_{y}^{2}\left(x{\beta }_{y}+y(\gamma -1)\delta {\beta }_{x}\right)}{\mathop{{\eta }_{1}^{3}}\limits_{z}}\,\end{array}\right)\\ {({r}^{\prime\prime\prime })}^{\ell t} & = & A{(\Delta \overrightarrow{\beta })}^{\ell t}\cdot A{(\overrightarrow{\beta })}^{\ell t}\cdot {(r)}^{\ell t}=A{(\Delta \overrightarrow{\beta })}^{\ell t}\cdot {({r}^{{\prime} })}^{\ell t}\\  & = & \left(\begin{array}{c}\gamma \left(-\frac{\left(x{\beta }_{x}+y{\beta }_{y}\right)\left({\beta }_{x}\delta {\beta }_{x}+{\beta }_{y}\delta {\beta }_{y}\right){\gamma }^{2}}{{\eta }_{1}^{2}}-x{\beta }_{x}-y{\beta }_{y}-\frac{\left(y{\beta }_{x}-x{\beta }_{y}\right){\lambda }_{6}}{{\eta }_{1}^{2}}\right)\\ \frac{\gamma \left(x{\beta }_{x}+y{\beta }_{y}\right)\left({\beta }_{x}\delta {\beta }_{x}{\gamma }^{2}+{\beta }_{y}\delta {\beta }_{y}{\gamma }^{2}+1\right)}{{\eta }_{1}}\\ \frac{x{\gamma }^{2}\delta {\beta }_{y}{\beta }_{x}^{2}+\left({\beta }_{y}\left(y\delta {\beta }_{y}-x\delta {\beta }_{x}\right){\gamma }^{2}+y\right){\beta }_{x}-{\beta }_{y}\left(y{\beta }_{y}\delta {\beta }_{x}{\gamma }^{2}+x\right)}{{\eta }_{1}}\\ z\end{array}\right)\end{array}$$

One way to check if Eqs. (45) and (46) are correct is to show that the invariant interval *ds*^2^^16^:

47 $$d{s}^{2}={g}_{\mu \nu }d{x}^{\mu }d{x}^{\nu }={(d{x}^{0})}^{2}-{(d{x}^{1})}^{2}-{(d{x}^{2})}^{2}-{(d{x}^{3})}^{2}$$

remains the same, where *g*_*μν*_ is the metric tensor:

$${g}_{\mu \nu }=\left(\begin{array}{cccc}1 & 0 & 0 & 0\\ 0 & -1 & 0 & 0\\ 0 & 0 & -1 & 0\\ 0 & 0 & 0 & -1\end{array}\right)$$

In our case we are concerned with the invariance of

48 $${s}^{2}={x}_{0}^{2}-{x}_{1}^{2}-{x}_{2}^{2}-{x}_{3}^{2}$$

To see if that is the case, we can apply Eq. (48) to ${({r}^{{\prime} })}^{\ell t}$, (*r*″)^*ℓt*^ and ${({r}^{\prime\prime\prime })}^{\ell t}$ calculated above. The invariant

$${s}^{2}=-{x}^{2}-{y}^{2}-{z}^{2}$$

indeed stays the same for each case. This makes sense since we ignored the time component.

Similar results can be obtained for the position 4-vector *r* in the lab *xy* -frame and it can be easily proved that the invariant does not change. We also compare our approach with Ungar’s in the Supplementary Information (Section 4).

## Conclusion

The work presented in this paper is another confirmation of the fact that two successive boosts are not equal to a single direct boost. In the case of the electromagnetic field, just applying the usual electromagnetic field transformation equations will not result in the correct form of electromagnetic fields in the case of non-collinear boosts (accelerating frames) as Thomas rotation must be included.

Apart from the validations made in the previous section we will see if the electromagnetic field tensors in the direct boosted frame $\overrightarrow{\beta }+\delta \overrightarrow{\beta }$ and the successively boosted frames $\overrightarrow{\beta }$ and $\Delta \overrightarrow{\beta }$ are consistent with the Thomas rotation. To see that we can take the difference between the corresponding elements of *F*″ and *F*‴ in both the longitudinal-transverse *ℓt* and lab *xy*-frames.

After taking the difference of the electromagnetic field tensors *F″* and *F*‴ we found that

49 $${({F}^{{\prime\prime} })}_{ij}-{({F}^{\prime\prime\prime })}_{ij}\propto (\gamma -1)$$

for both *ℓt*- and *xy*-frames. This makes sense because both *F″* and *F*‴ just differ by Thomas rotation. Although taking the difference of *F″* and *F*‴ is not very significant physically it does show what we expected.

To our knowledge, this is the first time that someone has calculated the expressions of the electromagnetic fields in the frames corresponding to general three-dimensional non-collinear boosts.

One application of this work concerns the calculation of shifts in the Larmor frequency of highly relativistic particles moving through non-uniform magnetic and electric fields. Such a formalism was developed for the motion of non-relativistic particles^17–19^; however, this formalism is not directly applicable to relativistic particles because the formalism assumes the electromagnetic fields are known in the particle rest frame. For a highly relativistic particle undergoing acceleration (e.g., relativistic charged particles stored by electromagnetic fields within a circular storage ring), one can then apply the formalism developed here in this paper to determine the electromagnetic fields in an appropriate reference frame, where any residual motion of the particle is then non-relativistic, and then proceed to calculate the frequency shifts per the formalism of^17–19^.

## Supplementary information

Supplementary information.
