# Supplementary material for: Effect of Thomas Rotation on the Lorentz Transformation of Electromagnetic fields
Source: Sci Rep. 2020 Mar 26;10:5522. doi: 10.1038/s41598-020-62082-z (PMC7099098; doi:10.1038/s41598-020-62082-z)
Supplement: Supplementary file 1 — Supplementary information. [file 41598_2020_62082_MOESM1_ESM.pdf]

Effect of Thomas Rotation on the Lorentz Transformation of Electromagnetic fields

Lakshya Malhotra[1,*], **Robert Golub**[2], **Eva Kraegeloh**[3], **Nima Nouri**[1,+], **and Bradley Plaster**[1]

[1]University of Kentucky, Department of Physics and Astronomy, Lexington, Kentucky, 40508, USA
[2]North Carolina State University,Department of Physics, Raleigh, North Carolina, 27695, USA
[3]University of Michigan, Department of Physics, Ann Arbor, Michigan, 48109, USA
[*]lakshya.malhotra@uky.edu
[+]Present Address: Yale University School of Medicine, Department of Pathology, New Haven, CT, 06510, USA

ABSTRACT

This is the supplementary information for the main article. It includes the mathematical equations for all the electromagnetic fields in different reference frames.

1 Notation

We begin by recalling that the electromagnetic field tensor $F^{\mu\nu}$ is an anti-symmetric matrix:

$$F^{\mu\nu} = \begin{pmatrix} 0 & -E_x & -E_y & -E_z \\ E_x & 0 & -B_z & B_y \\ E_y & B_z & 0 & -B_x \\ E_z & -B_y & B_x & 0 \end{pmatrix} \tag{1}$$

Defining some dummy variables to make equations look cleaner:

$$\begin{aligned}
\lambda_1 &= \sqrt{\beta_x^2+\beta_y^2+\beta_z^2} \\
\lambda_2 &= \beta_x\delta\beta_x+\beta_y\delta\beta_y+\beta_z\delta\beta_z \\
\lambda_3 &= \beta_y^2\delta\beta_x-\beta_x\beta_y\delta\beta_y+\beta_z\left(\beta_z\delta\beta_x-\beta_x\delta\beta_z\right) \\
\lambda_4 &= \beta_x^2\delta\beta_y-\beta_x\beta_y\delta\beta_x+\beta_z\left(\beta_z\delta\beta_y-\beta_y\delta\beta_z\right) \\
\lambda_5 &= \beta_x^2\delta\beta_z-\beta_x\beta_z\delta\beta_x+\beta_y\left(\beta_y\delta\beta_z-\beta_z\delta\beta_y\right) \\
\lambda_6 &= \beta_x\delta\beta_y-\beta_y\delta\beta_x \\
\lambda_7 &= \beta_y\delta\beta_z-\beta_z\delta\beta_y \\
\lambda_8 &= \beta_z\delta\beta_x-\beta_x\delta\beta_z \\
\eta_1 &= \sqrt{\beta_x^2+\beta_y^2} \\
\eta_2 &= \sqrt{\beta_y^2+\beta_z^2} \\
\eta_3 &= \sqrt{\beta_x^2+\beta_z^2}
\end{aligned}$$

The detailed expressions of the electromagnetic fields (to the first order in $\delta\beta$) is given in the next two sections.

2 Longitudinal-Transverse ℓt-Frame

2.1 Direct Boosted Frame $(\vec{\beta} + \delta\vec{\beta})$

$$
\begin{aligned}
(F'')^{10} = \frac{1}{\lambda_1}\Bigg[& \beta_x E_x + \beta_y E_y + \beta_z E_z - \lambda_3 E_x \gamma^2 - \lambda_4 E_y \gamma^2 - \lambda_5 E_z \gamma^2 + B_z \lambda_6 \gamma + B_y \lambda_8 \gamma + B_x \lambda_7 \gamma + \frac{\gamma(\gamma-1)\eta_2^2 \delta\beta_x E_x}{\lambda_1^2} \\
& + \frac{\gamma(\gamma-1)\eta_3^2 \delta\beta_y E_y}{\lambda_1^2} + \frac{\gamma(\gamma-1)\eta_1^2 \delta\beta_z E_z}{\lambda_1^2} - \frac{\gamma(\gamma-1)\beta_x\beta_y\delta\beta_y E_x}{\lambda_1^2} - \frac{\gamma(\gamma-1)\beta_x\beta_z\delta\beta_z E_x}{\lambda_1^2} - \frac{\gamma(\gamma-1)\beta_x\beta_y\delta\beta_x E_y}{\lambda_1^2} \\
& - \frac{\gamma(\gamma-1)\beta_y\beta_z\delta\beta_z E_y}{\lambda_1^2} - \frac{\gamma(\gamma-1)\beta_x\beta_z\delta\beta_x E_z}{\lambda_1^2} - \frac{\gamma(\gamma-1)\beta_y\beta_z\delta\beta_y E_z}{\lambda_1^2}\Bigg]
\end{aligned}
$$

$$
\begin{aligned}
(F'')^{20} = & -\gamma B_z \eta_1 + \frac{\gamma B_x \beta_x \beta_z}{\eta_1} + \frac{\gamma B_y \beta_y \beta_z}{\eta_1} - \frac{\gamma E_x \beta_y}{\eta_1} + \frac{\gamma \beta_x E_y}{\eta_1} - \frac{\gamma^3 B_z \beta_x \eta_1 \delta\beta_x}{\lambda_1^2} + \frac{\gamma^2 \delta\beta_x \beta_y \beta_z E_z}{\eta_1} - \frac{\gamma^3 B_z \beta_y \eta_1 \delta\beta_y}{\lambda_1^2} \\
& + \frac{\gamma\left(\gamma^2-1\right) B_x \beta_x^2 \beta_z \delta\beta_x}{\eta_1 \lambda_1^2} + \frac{\gamma\left(\gamma^2-1\right) B_y \beta_x \delta\beta_x \beta_y \beta_z}{\eta_1 \lambda_1^2} - \frac{\gamma B_z \beta_x \delta\beta_x \beta_z^2}{\eta_1 \lambda_1^2} - \frac{\gamma B_z \beta_y \delta\beta_y \beta_z^2}{\eta_1 \lambda_1^2} + \frac{\gamma B_x \beta_x \eta_1 \delta\beta_z}{\lambda_1^2} \\
& + \frac{\gamma\left(\gamma^2-1\right) B_y \beta_y^2 \delta\beta_y \beta_z}{\eta_1 \lambda_1^2} + \frac{\gamma\left(\gamma^2-1\right) B_x \beta_x \beta_y \delta\beta_y \beta_z}{\eta_1 \lambda_1^2} - \frac{\gamma\left(\gamma^2-1\right) B_z \beta_z \eta_1 \delta\beta_z}{\lambda_1^2} - \frac{(\gamma-1)\gamma^2 \beta_x \delta\beta_x E_x \beta_y}{\eta_1} \\
& + \frac{\gamma^3 B_x \beta_x \beta_z^2 \delta\beta_z}{\eta_1 \lambda_1^2} + \frac{\gamma^3 B_y \beta_y \beta_z^2 \delta\beta_z}{\eta_1 \lambda_1^2} + \frac{\gamma B_y \beta_y \eta_1 \delta\beta_z}{\lambda_1^2} - \frac{\gamma^3 E_x \beta_y^2 \delta\beta_y}{\eta_1} - \frac{\gamma^2 \beta_x^2 E_x \delta\beta_y}{\eta_1} - \frac{\gamma^3 E_x \beta_y \beta_z \delta\beta_z}{\eta_1} + \frac{\gamma^3 \beta_x^2 \delta\beta_x E_y}{\eta_1} \\
& + \frac{(\gamma-1)\gamma\beta_x^2 E_x \delta\beta_y}{\eta_1 \lambda_1^2} - \frac{(\gamma-1)\gamma\beta_x \delta\beta_x E_x \beta_y}{\eta_1 \lambda_1^2} + \frac{(\gamma-1)\gamma^2 \beta_x \beta_y \delta\beta_y E_y}{\eta_1} - \frac{(\gamma-1)\gamma\delta\beta_x \beta_y^2 E_y}{\eta_1 \lambda_1^2} + \frac{\gamma^2 \delta\beta_x \beta_y^2 E_y}{\eta_1} \\
& + \frac{\gamma^3 \beta_x E_y \beta_z \delta\beta_z}{\eta_1} - \frac{\gamma^2 \beta_x \delta\beta_y \beta_z E_z}{\eta_1} + \frac{(\gamma-1)\gamma\beta_x \delta\beta_y \beta_z E_z}{\eta_1 \lambda_1^2} - \frac{(\gamma-1)\gamma\delta\beta_x \beta_y \beta_z E_z}{\eta_1 \lambda_1^2} + \frac{(\gamma-1)\gamma\beta_x \beta_y \delta\beta_y E_y}{\eta_1 \lambda_1^2}
\end{aligned}
$$

$$
\begin{aligned}
(F'')^{30} = & \frac{\gamma B_y \beta_x \lambda_1}{\eta_1} - \frac{\gamma B_x \beta_y \lambda_1}{\eta_1} - \frac{\gamma \beta_x E_x \beta_z}{\eta_1 \lambda_1} - \frac{\gamma \beta_y E_y \beta_z}{\eta_1 \lambda_1} + \frac{\gamma E_z \eta_1}{\lambda_1} + \frac{\gamma^3 B_y \beta_x^2 \delta\beta_x}{\eta_1 \lambda_1} + \frac{\gamma B_y \delta\beta_x \beta_y^2}{\eta_1 \lambda_1} - \frac{\gamma B_x \beta_x^2 \delta\beta_y}{\eta_1 \lambda_1} \\
& - \frac{\gamma^3 B_x \beta_y^2 \delta\beta_y}{\eta_1 \lambda_1} - \frac{\gamma B_z \beta_z \lambda_6}{\eta_1 \lambda_1} + \frac{\gamma\left(\gamma^2-1\right) B_y \beta_x \beta_y \delta\beta_y}{\eta_1 \lambda_1} - \frac{\gamma\left(\gamma^2-1\right) B_x \beta_x \delta\beta_x \beta_y}{\eta_1 \lambda_1} - \frac{(\gamma-1)\gamma\beta_x^2 \delta\beta_x E_x \beta_z}{\eta_1 \lambda_1^3} \\
& + \frac{\gamma^3 B_y \beta_x \beta_z \delta\beta_z}{\eta_1 \lambda_1} - \frac{\gamma^3 B_x \beta_y \beta_z \delta\beta_z}{\eta_1 \lambda_1} - \frac{\gamma^2 \beta_x E_x \delta\beta_z \eta_1}{\lambda_1} - \frac{(\gamma-1)\gamma^2 \beta_x^2 \delta\beta_x E_x \beta_z}{\eta_1 \lambda_1} - \frac{(\gamma-1)\gamma^2 \beta_x E_x \beta_y \delta\beta_y \beta_z}{\eta_1 \lambda_1} \\
& + \frac{(\gamma-1)\gamma\beta_x E_x \delta\beta_z \eta_1}{\lambda_1^3} - \frac{(\gamma-1)\gamma\beta_x E_x \beta_y \delta\beta_y \beta_z}{\eta_1 \lambda_1^3} - \frac{(\gamma-1)\gamma^2 \beta_x \delta\beta_x \beta_y E_y \beta_z}{\eta_1 \lambda_1} - \frac{(\gamma-1)\gamma^2 \beta_y^2 \delta\beta_y E_y \beta_z}{\eta_1 \lambda_1} \\
& - \frac{\gamma^3 \beta_x E_x \beta_z^2 \delta\beta_z}{\eta_1 \lambda_1} - \frac{\gamma^2 \beta_y E_y \delta\beta_z \eta_1}{\lambda_1} + \frac{(\gamma-1)\gamma\beta_y E_y \lambda_5}{\eta_1 \lambda_1^3} - \frac{\gamma^3 \beta_y E_y \beta_z^2 \delta\beta_z}{\eta_1 \lambda_1} + \frac{(\gamma-1)\gamma\beta_z \delta\beta_z E_z \eta_1}{\lambda_1^3} \\
& + \frac{\gamma^3 \beta_y \delta\beta_y E_z \eta_1}{\lambda_1} + \frac{\gamma^3 \beta_x \delta\beta_x E_z \eta_1}{\lambda_1} + \frac{\gamma^2 \beta_x \delta\beta_x \beta_z^2 E_z}{\eta_1 \lambda_1} - \frac{(\gamma-1)\gamma\beta_y \delta\beta_y \beta_z^2 E_z}{\eta_1 \lambda_1^3} + \frac{(\gamma-1)\gamma^2 \beta_z \delta\beta_z E_z \eta_1^3}{\lambda_1^3} \\
& + \frac{\gamma^2 \beta_y \delta\beta_y \beta_z^2 E_z}{\eta_1 \lambda_1} + \frac{(\gamma-1)\gamma^2 \beta_z^3 \delta\beta_z E_z \eta_1}{\lambda_1^3} - \frac{(\gamma-1)\gamma\beta_x \delta\beta_x \beta_z^2 E_z}{\eta_1 \lambda_1^3}
\end{aligned}
$$

$$
(F'')^{32} = \frac{1}{\lambda_1}\left[B_x\beta_x + B_y\beta_y + B_z\beta_z - \gamma E_x \lambda_7 - \gamma E_y \lambda_8 - \gamma E_z \lambda_6 - \frac{(\gamma-1)B_z\lambda_5}{\lambda_1^2} - \frac{(\gamma-1)B_y\lambda_4}{\lambda_1^2} - \frac{(\gamma-1)B_x\lambda_3}{\lambda_1^2}\right]
$$

$$
\begin{aligned}
(F'')^{13} = & -\frac{\gamma B_x\beta_y}{\eta_1}+\frac{\gamma B_y\beta_x}{\eta_1}-\frac{\gamma\beta_x E_x\beta_z}{\eta_1}-\frac{\gamma\beta_y E_y\beta_z}{\eta_1}+\gamma E_z\eta_1-\frac{\gamma^3 B_x\beta_x\delta\beta_x\beta_y}{\eta_1}-\frac{\gamma^3 B_x\beta_y^2\delta\beta_y}{\eta_1}-\frac{\gamma^3 B_x\beta_y\beta_z\delta\beta_z}{\eta_1} \\
& +\frac{\gamma^3 B_y\beta_x\beta_y\delta\beta_y}{\eta_1}+\frac{\gamma^3 B_y\beta_x^2\delta\beta_x}{\eta_1}+\frac{\gamma^3 B_y\beta_x\beta_z\delta\beta_z}{\eta_1}-\frac{\gamma^3\beta_x E_x\beta_z^2\delta\beta_z}{\eta_1\lambda_1^2}+\frac{(\gamma-1)B_x\beta_x\delta\beta_x\beta_y}{\eta_1\lambda_1^2}-\frac{(\gamma-1)B_x\beta_x^2\delta\beta_y}{\eta_1\lambda_1^2} \\
& +\frac{(\gamma-1)B_y\delta\beta_x\beta_y^2}{\eta_1\lambda_1^2}+\frac{(\gamma-1)B_z\delta\beta_x\beta_y\beta_z}{\eta_1\lambda_1^2}-\frac{(\gamma-1)B_y\beta_x\beta_y\delta\beta_y}{\eta_1\lambda_1^2}-\frac{(\gamma-1)B_z\beta_x\delta\beta_y\beta_z}{\eta_1\lambda_1^2}-\frac{\left(\gamma^2-1\right)\gamma\beta_x^2\delta\beta_x E_x\beta_z}{\eta_1\lambda_1^2} \\
& -\frac{\left(\gamma^2-1\right)\gamma\beta_x E_x\beta_y\delta\beta_y\beta_z}{\eta_1\lambda_1^2}-\frac{\gamma\beta_x E_x\delta\beta_z\eta_1}{\lambda_1^2}-\frac{\gamma\left(\gamma^2-1\right)\beta_y^2\delta\beta_y E_y\beta_z}{\eta_1\lambda_1^2}-\frac{\gamma\left(\gamma^2-1\right)\beta_x\delta\beta_x\beta_y E_y\beta_z}{\eta_1\lambda_1^2}-\frac{\gamma\beta_y E_y\delta\beta_z\eta_1}{\lambda_1^2} \\
& -\frac{\gamma^3\beta_y E_y\beta_z^2\delta\beta_z}{\eta_1\lambda_1^2}+\frac{\gamma^3\beta_y\delta\beta_y E_z\eta_1}{\lambda_1^2}+\frac{\gamma^3\beta_x\delta\beta_x E_z\eta_1}{\lambda_1^2}+\frac{\gamma\beta_x\delta\beta_x\beta_z^2 E_z}{\eta_1\lambda_1^2}+\frac{\gamma\left(\gamma^2-1\right)\beta_z\delta\beta_z E_z\eta_1}{\lambda_1^2}+\frac{\gamma\beta_y\delta\beta_y\beta_z^2 E_z}{\eta_1\lambda_1^2}
\end{aligned}
$$

$$
\begin{aligned}
(F'')^{12} = & \frac{\gamma B_z\eta_1}{\lambda_1}-\frac{\gamma B_x\beta_x\beta_z}{\eta_1\lambda_1}-\frac{\gamma B_y\beta_y\beta_z}{\eta_1\lambda_1}+\frac{\gamma E_x\beta_y\lambda_1}{\eta_1}-\frac{\gamma\beta_x E_y\lambda_1}{\eta_1}+\frac{(\gamma-1)B_x\beta_x^2\delta\beta_x\beta_z}{\eta_1\lambda_1^3}-\frac{\gamma^3 B_x\beta_x^2\delta\beta_x\beta_z}{\eta_1\lambda_1}-\frac{\gamma\delta\beta_x\beta_y^2 E_y}{\eta_1\lambda_1} \\
& -\frac{\gamma^3 B_y\beta_x\delta\beta_x\beta_y\beta_z}{\eta_1\lambda_1}+\frac{\gamma^3 B_z\beta_x\delta\beta_x\eta_1}{\lambda_1}+\frac{\gamma^3 B_z\beta_y\delta\beta_y\eta_1}{\lambda_1}-\frac{\gamma^3 B_x\beta_x\beta_y\delta\beta_y\beta_z}{\eta_1\lambda_1}-\frac{\gamma^3 B_y\beta_y^2\delta\beta_y\beta_z}{\eta_1\lambda_1}-\frac{\gamma^3 B_x\beta_x\beta_z^2\delta\beta_z}{\eta_1\lambda_1} \\
& +\frac{(\gamma-1)B_z\beta_x\delta\beta_x\beta_z^2}{\eta_1\lambda_1^3}+\frac{(\gamma-1)B_y\beta_x\delta\beta_x\beta_y\beta_z}{\eta_1\lambda_1^3}+\frac{(\gamma-1)B_x\beta_x\beta_y\delta\beta_y\beta_z}{\eta_1\lambda_1^3}+\frac{(\gamma-1)B_y\beta_y^2\delta\beta_y\beta_z}{\eta_1\lambda_1^3}+\frac{(\gamma-1)B_z\beta_y\delta\beta_y\beta_z^2}{\eta_1\lambda_1^3} \\
& -\frac{(\gamma-1)B_x\beta_x\delta\beta_z\eta_1}{\lambda_1^3}-\frac{(\gamma-1)B_y\beta_y\delta\beta_z\eta_1}{\lambda_1^3}-\frac{(\gamma-1)B_z\beta_z\delta\beta_z\eta_1}{\lambda_1^3}+\frac{\gamma\left(\gamma^2-1\right)\beta_x\delta\beta_x E_x\beta_y}{\eta_1\lambda_1}-\frac{\left(\gamma^2-1\right)\gamma\beta_x\beta_y\delta\beta_y E_y}{\eta_1\lambda_1} \\
& -\frac{\gamma^3 B_y\beta_y\beta_z^2\delta\beta_z}{\eta_1\lambda_1}+\frac{\gamma^3 B_z\beta_z\delta\beta_z\eta_1}{\lambda_1}+\frac{\gamma^3 E_x\beta_y^2\delta\beta_y}{\eta_1\lambda_1}+\frac{\gamma^3 E_x\beta_y\beta_z\delta\beta_z}{\eta_1\lambda_1}+\frac{\gamma\beta_x^2 E_x\delta\beta_y}{\eta_1\lambda_1}-\frac{\gamma^3\beta_x^2\delta\beta_x E_y}{\eta_1\lambda_1}-\frac{\gamma^3\beta_x E_y\beta_z\delta\beta_z}{\eta_1\lambda_1} \\
& -\frac{\gamma\delta\beta_x\beta_y\beta_z E_z}{\eta_1\lambda_1}+\frac{\gamma\beta_x\delta\beta_y\beta_z E_z}{\eta_1\lambda_1}
\end{aligned}
$$

2.2 Successively Boosted Frame $\vec{\beta}$ and $\Delta\vec{\beta}$

$$
\begin{aligned}
(F''')^{10} = & \frac{\beta_x E_x}{\lambda_1}+\frac{\beta_y E_y}{\lambda_1}+\frac{\beta_z E_z}{\lambda_1}+\frac{\gamma^2 B_z\lambda_6}{\lambda_1}+\frac{\gamma^2 B_y\lambda_8}{\lambda_1}+\frac{\gamma^2 B_x\lambda_7}{\lambda_1}+\frac{\gamma^2\beta_x E_x\beta_y\delta\beta_y}{\lambda_1}+\frac{\gamma^2\beta_x E_x\beta_z\delta\beta_z}{\lambda_1}-\frac{\gamma^2\delta\beta_x E_x\eta_2^2}{\lambda_1} \\
& +\frac{\gamma^2\beta_y E_y\beta_z\delta\beta_z}{\lambda_1}-\frac{\gamma^2\delta\beta_y E_y\eta_3^2}{\lambda_1}+\frac{\gamma^2\beta_x\delta\beta_x\beta_y E_y}{\lambda_1}+\frac{\gamma^2\beta_y\delta\beta_y\beta_z E_z}{\lambda_1}+\frac{\gamma^2\beta_x\delta\beta_x\beta_z E_z}{\lambda_1}-\frac{\gamma^2\delta\beta_z E_z\eta_1^2}{\lambda_1}
\end{aligned}
$$

$$
\begin{aligned}
(F''')^{20} = & \frac{2\gamma B_y\beta_y\beta_z}{\eta_1}-\gamma B_z\eta_1-\frac{\gamma E_x\beta_y}{\eta_1}+\frac{\gamma\beta_x E_y}{\eta_1}-\frac{\gamma^3 B_z\eta_1\lambda_2}{\lambda_1^2}+\frac{\gamma B_z\beta_z\lambda_5}{\eta_1\lambda_1^2}-\frac{\gamma^3 E_x\beta_y\lambda_2}{\eta_1}+\frac{\gamma^3\beta_x E_y\lambda_2}{\eta_1}+\frac{2\gamma B_y\beta_y\delta\beta_z\eta_1}{\lambda_1^2} \\
& +\frac{2\gamma\left(\gamma^2-1\right)B_y\beta_y^2\delta\beta_y\beta_z}{\eta_1\lambda_1^2}+\frac{2\gamma\left(\gamma^2-1\right)B_y\beta_x\delta\beta_x\beta_y\beta_z}{\eta_1\lambda_1^2}+\frac{2\gamma^3 B_y\beta_y\beta_z^2\delta\beta_z}{\eta_1\lambda_1^2}
\end{aligned}
$$

$$
\begin{aligned}
(F''')^{30} = & \frac{\gamma B_y\beta_x\lambda_1}{\eta_1}-\frac{\gamma B_x\beta_y\lambda_1}{\eta_1}-\frac{\gamma\beta_x E_x\beta_z}{\eta_1\lambda_1}-\frac{\gamma\beta_y E_y\beta_z}{\eta_1\lambda_1}+\frac{\gamma E_z\eta_1}{\lambda_1}-\frac{\gamma B_x\beta_x\lambda_6}{\eta_1\lambda_1}-\frac{\gamma B_y\beta_y\lambda_6}{\eta_1\lambda_1}-\frac{\gamma B_z\beta_z\lambda_6}{\eta_1\lambda_1}+\frac{\gamma^3 B_y\beta_x\lambda_2}{\eta_1\lambda_1} \\
& -\frac{\gamma^3 B_x\beta_y\lambda_2}{\eta_1\lambda_1}-\frac{\gamma^3\beta_x E_x\beta_z\lambda_2}{\eta_1\lambda_1}-\frac{\gamma^3\beta_y E_y\beta_z\lambda_2}{\eta_1\lambda_1}+\frac{\gamma^3 E_z\eta_1\lambda_2}{\lambda_1}
\end{aligned}
$$

$$
\begin{aligned}
(F''')^{32} = & \frac{1}{\lambda_1}\left[B_x\beta_x+B_y\beta_y+B_z\beta_z-\gamma^2 E_x\lambda_7-\gamma^2 E_y\lambda_8-\gamma^2 E_z\lambda_6+\gamma^2 B_z\beta_x\delta\beta_x\beta_z+\gamma^2 B_x\beta_x\beta_y\delta\beta_y+\gamma^2 B_z\beta_y\delta\beta_y\beta_z\right. \\
& \left.-\gamma^2 B_x\delta\beta_x\eta_2^2-\gamma^2 B_y\delta\beta_y\eta_3^2-\gamma^2 B_z\delta\beta_z\eta_1^2+\gamma^2 B_y\beta_y\beta_z\delta\beta_z+\gamma^2 B_x\beta_x\beta_z\delta\beta_z+\gamma^2 B_y\beta_x\delta\beta_x\beta_y\right]
\end{aligned}
$$

$$(F''')^{13} = -\frac{\gamma B_x\beta_y}{\eta_1} + \frac{\gamma B_y\beta_x}{\eta_1} - \frac{\gamma\beta_x E_x\beta_z}{\eta_1} - \frac{\gamma\beta_y E_y\beta_z}{\eta_1} + \gamma E_z\eta_1 - \frac{\gamma^3 B_x\beta_y\lambda_2}{\eta_1} + \frac{\gamma^3 B_y\beta_x\lambda_2}{\eta_1} - \frac{\gamma^3\beta_x E_x\beta_z\lambda_2}{\eta_1\lambda_1^2} - \frac{\gamma^3\beta_y E_y\beta_z\lambda_2}{\eta_1\lambda_1^2}$$
$$- \frac{\gamma\beta_x E_x\lambda_5}{\eta_1\lambda_1^2} - \frac{\gamma\beta_y E_y\lambda_5}{\eta_1\lambda_1^2} + \frac{\gamma^3 E_z\eta_1\lambda_2}{\lambda_1^2} - \frac{\gamma\beta_z E_z\lambda_5}{\eta_1\lambda_1^2}$$

$$(F''')^{21} = \frac{\gamma B_z\eta_1}{\lambda_1} - \frac{\gamma B_x\beta_x\beta_z}{\eta_1\lambda_1} - \frac{\gamma B_y\beta_y\beta_z}{\eta_1\lambda_1} + \frac{\gamma E_x\beta_y\lambda_1}{\eta_1} - \frac{\gamma\beta_x E_y\lambda_1}{\eta_1} - \frac{\gamma^3 B_x\beta_x\beta_z\lambda_2}{\eta_1\lambda_1} - \frac{\gamma^3 B_y\beta_y\beta_z\lambda_2}{\eta_1\lambda_1} + \frac{\gamma^3 B_z\eta_1\lambda_2}{\lambda_1} + \frac{\gamma^3 E_x\beta_y\lambda_2}{\eta_1\lambda_1}$$
$$+ \frac{\gamma\beta_x E_x\lambda_6}{\eta_1\lambda_1} - \frac{\gamma^3\beta_x E_y\lambda_2}{\eta_1\lambda_1} + \frac{\gamma\beta_y E_y\lambda_6}{\eta_1\lambda_1} + \frac{\gamma\beta_z E_z\lambda_6}{\eta_1\lambda_1}$$

3 Laboratory xy-Frame

3.1 Direct Boosted Frame $(\vec{\beta} + \delta\vec{\beta})$

$$(F'')^{10} = \gamma B_z\beta_y - \gamma B_y\beta_z - \gamma^2\beta_x^2 E_x + \frac{1}{\lambda_1^2}\left[\gamma E_x\left(\gamma\beta_x^2 + \beta_y^2 + \beta_z^2\right) - (\gamma-1)\beta_x\beta_y E_y - (\gamma-1)\beta_x\beta_z E_z - \gamma^3 B_y\beta_z\lambda_2 + \gamma^3 B_z\beta_y\lambda_2\right.$$
$$+\gamma B_z\lambda_4 + \gamma^3\beta_x\delta\beta_x E_x\eta_2^2 + \gamma^3 E_x\beta_y\delta\beta_y\eta_2^2 + \gamma^3 E_x\beta_z\delta\beta_z\eta_2^2 - \gamma^3\beta_x\beta_y\delta\beta_y\beta_z E_z - \gamma^3\beta_x\beta_z^2\delta\beta_z E_z - \gamma^3\beta_x^2\delta\beta_x\beta_y E_y$$
$$-\gamma B_y\lambda_5 - \gamma^3\beta_x\beta_y^2\delta\beta_y E_y - \gamma^3\beta_x\beta_y E_y\beta_z\delta\beta_z - \gamma^3\beta_x^2\delta\beta_x\beta_z E_z + \frac{2(\gamma-1)\beta_x^2 E_x\beta_y\delta\beta_y}{\lambda_1^2} + \frac{2(\gamma-1)\beta_x^2 E_x\beta_z\delta\beta_z}{\lambda_1^2}$$
$$-\frac{(\gamma-1)\delta\beta_x\beta_y E_y\left(-\beta_x^2+\beta_y^2+\beta_z^2\right)}{\lambda_1^2} + \frac{2(\gamma-1)\beta_x\beta_y E_y\beta_z\delta\beta_z}{\lambda_1^2} - \frac{(\gamma-1)\beta_x\delta\beta_y E_y\left(\beta_x^2-\beta_y^2+\beta_z^2\right)}{\lambda_1^2} + \frac{2(\gamma-1)\beta_x\beta_y\delta\beta_y\beta_z E_z}{\lambda_1^2}$$
$$\left.-\frac{(\gamma-1)\delta\beta_x\beta_z E_z\left(-\beta_x^2+\beta_y^2+\beta_z^2\right)}{\lambda_1^2} - \frac{(\gamma-1)\beta_x\delta\beta_z E_z\left(\beta_x^2+\beta_y^2-\beta_z^2\right)}{\lambda_1^2} - \frac{2(\gamma-1)\beta_x\delta\beta_x E_x\eta_2^2}{\lambda_1^2}\right]$$

$$(F'')^{20} = \gamma B_x\beta_z - \gamma B_z\beta_x - \gamma^2\beta_x E_x\beta_y - \gamma^2\beta_y^2 E_y - \gamma^2\beta_y\beta_z E_z + \frac{1}{\lambda_1^2}\left[\gamma^2\beta_y^2 E_y + \gamma E_y\eta_3^2 + \gamma^3\beta_x\delta\beta_x E_y\eta_3^2 + \gamma^3\beta_y\delta\beta_y E_y\eta_3^2\right.$$
$$+(\gamma-1)\gamma\beta_x E_x\beta_y + (\gamma-1)\gamma\beta_y\beta_z E_z - \gamma^3 B_z\beta_x^2\delta\beta_x + \left(\gamma^2-1\right)\gamma B_x\beta_x\delta\beta_x\beta_z - \left(\gamma^2-1\right)\gamma B_z\beta_x\beta_y\delta\beta_y - \gamma B_z\delta\beta_x\eta_2^2$$
$$+\gamma^3 B_x\beta_z^2\delta\beta_z - \left(\gamma^2-1\right)\gamma B_z\beta_x\beta_z\delta\beta_z + \gamma\left(\gamma^2-1\right)B_x\beta_y\delta\beta_y\beta_z + \gamma B_x\delta\beta_z\eta_1^2 - \gamma^3\beta_x E_x\beta_y\beta_z\delta\beta_z + \gamma^3 E_y\beta_z\delta\beta_z\eta_3^2$$
$$-\gamma^3\beta_x^2\delta\beta_x E_x\beta_y - \gamma^3\beta_x E_x\beta_y^2\delta\beta_y - \gamma^3\beta_x\delta\beta_x\beta_y\beta_z E_z - \gamma^3\beta_y^2\delta\beta_y\beta_z E_z - \gamma^3\beta_y\beta_z^2\delta\beta_z E_z - \frac{(\gamma-1)\beta_y\delta\beta_z E_z\left(\beta_x^2+\beta_y^2-\beta_z^2\right)}{\lambda_1^2}$$
$$-\frac{(\gamma-1)\delta\beta_x E_x\beta_y\left(-\beta_x^2+\beta_y^2+\beta_z^2\right)}{\lambda_1^2} - \frac{(\gamma-1)\beta_x E_x\delta\beta_y\left(\beta_x^2-\beta_y^2+\beta_z^2\right)}{\lambda_1^2} + \frac{2(\gamma-1)\beta_x\delta\beta_x\beta_y^2 E_y}{\lambda_1^2} - \frac{2(\gamma-1)\beta_y\delta\beta_y E_y\eta_3^2}{\lambda_1^2}$$
$$\left.+\frac{2(\gamma-1)\beta_y^2 E_y\beta_z\delta\beta_z}{\lambda_1^2} + \frac{2(\gamma-1)\beta_x\delta\beta_x\beta_y\beta_z E_z}{\lambda_1^2} - \frac{(\gamma-1)\delta\beta_y\beta_z E_z\left(\beta_x^2-\beta_y^2+\beta_z^2\right)}{\lambda_1^2} + \frac{2(\gamma-1)\beta_x E_x\beta_y\beta_z\delta\beta_z}{\lambda_1^2}\right]$$

$$(F'')^{30} = \gamma B_y\beta_x - \gamma B_x\beta_y - \gamma^2\beta_z^2 E_z + \frac{1}{\lambda_1^2}\left[\gamma^3 B_y\beta_x^2\delta\beta_x - (\gamma-1)\beta_x E_x\beta_z - (\gamma-1)\beta_y E_y\beta_z + \gamma E_z\left(\beta_x^2+\beta_y^2+\gamma\beta_z^2\right)\right.$$
$$+\gamma^3 B_y\beta_x\beta_y\delta\beta_y - \gamma^3 B_x\beta_y^2\delta\beta_y - \left(\gamma^2-1\right)\gamma B_x\beta_x\delta\beta_x\beta_y + \gamma B_y\delta\beta_x\eta_2^2 - \gamma B_y\beta_x\beta_y\delta\beta_y + \gamma^3 B_y\beta_x\beta_z\delta\beta_z$$
$$-\gamma^3 B_x\beta_y\beta_z\delta\beta_z + \gamma B_x\beta_y\beta_z\delta\beta_z - \gamma B_x\delta\beta_y\eta_3^2 - \gamma B_y\beta_x\beta_z\delta\beta_z - \gamma^3\beta_x^2\delta\beta_x E_x\beta_z - \gamma^3\beta_x E_x\beta_y\delta\beta_y\beta_z - \gamma^3\beta_y E_y\beta_z^2\delta\beta_z$$
$$-\gamma^3\beta_x E_x\beta_z^2\delta\beta_z - \gamma^3\beta_x\delta\beta_x\beta_y E_y\beta_z - \gamma^3\beta_y^2\delta\beta_y E_y\beta_z + \gamma^3\beta_x\delta\beta_x E_z\eta_1^2 + \gamma^3\beta_y\delta\beta_y E_z\eta_1^2 + \gamma^3\beta_z\delta\beta_z E_z\eta_1^2$$
$$-\frac{(\gamma-1)\delta\beta_x E_x\beta_z\left(-\beta_x^2+\beta_y^2+\beta_z^2\right)}{\lambda_1^2} + \frac{2(\gamma-1)\beta_x E_x\beta_y\delta\beta_y\beta_z}{\lambda_1^2} - \frac{(\gamma-1)\beta_x E_x\delta\beta_z\left(\beta_x^2+\beta_y^2-\beta_z^2\right)}{\lambda_1^2} + \frac{2(\gamma-1)\beta_x\delta\beta_x\beta_y E_y\beta_z}{\lambda_1^2}$$
$$-\frac{(\gamma-1)\delta\beta_y E_y\beta_z\left(\beta_x^2-\beta_y^2+\beta_z^2\right)}{\lambda_1^2} - \frac{(\gamma-1)\beta_y E_y\delta\beta_z\left(\beta_x^2+\beta_y^2-\beta_z^2\right)}{\lambda_1^2} + \frac{2(\gamma-1)\beta_x\delta\beta_x\beta_z^2 E_z}{\lambda_1^2} + \frac{2(\gamma-1)\beta_y\delta\beta_y\beta_z^2 E_z}{\lambda_1^2}$$
$$\left.-\frac{2(\gamma-1)\beta_z\delta\beta_z E_z\eta_1^2}{\lambda_1^2}\right]$$

$$(F'')^{32} = \gamma E_y\beta_z - \gamma\beta_y E_z + \frac{1}{\lambda_1^2}\left[B_x\beta_x^2 + \gamma B_x\eta_2^2 - (\gamma-1)B_y\beta_x\beta_y - (\gamma-1)B_z\beta_x\beta_z - \gamma^3 B_y\beta_x^2\delta\beta_x\beta_y - \gamma^3 B_z\beta_x^2\delta\beta_x\beta_z \right.$$
$$+\gamma^3 B_x\beta_x\delta\beta_x\eta_2^2 + \gamma^3 B_x\beta_y\delta\beta_y\eta_2^2 - \gamma^3 B_y\beta_x\beta_y^2\delta\beta_y - \gamma^3 B_z\beta_x\beta_y\delta\beta_y\beta_z + \gamma^3 B_x\beta_z\delta\beta_z\eta_2^2 - \gamma^3 B_z\beta_x\beta_z^2\delta\beta_z$$
$$-\gamma^3 B_y\beta_x\beta_y\beta_z\delta\beta_z - \gamma\delta\beta_y E_z\eta_3^2 + \gamma^3 E_y\beta_z^2\delta\beta_z + \left(\gamma^2-1\right)\gamma\beta_y\delta\beta_y E_y\beta_z + \gamma\left(\gamma^2-1\right)\beta_x\delta\beta_x E_y\beta_z + \gamma E_y\delta\beta_z\eta_1^2$$
$$-\gamma^3\beta_y^2\delta\beta_y E_z - \left(\gamma^2-1\right)\gamma\beta_x\delta\beta_x\beta_y E_z - \left(\gamma^2-1\right)\gamma\beta_y\beta_z\delta\beta_z E_z - \frac{2(\gamma-1)B_x\beta_x\delta\beta_x\eta_2^2}{\lambda_1^2} + \frac{2(\gamma-1)B_x\beta_x^2\beta_z\delta\beta_z}{\lambda_1^2}$$
$$+\frac{2(\gamma-1)B_y\beta_x\beta_y\beta_z\delta\beta_z}{\lambda_1^2} + \frac{2(\gamma-1)B_x\beta_x^2\beta_y\delta\beta_y}{\lambda_1^2} - \frac{(\gamma-1)B_z\delta\beta_x\beta_z\left(-\beta_x^2+\beta_y^2+\beta_z^2\right)}{\lambda_1^2} + \frac{2(\gamma-1)B_z\beta_x\beta_y\delta\beta_y\beta_z}{\lambda_1^2}$$
$$\left.-\frac{(\gamma-1)B_y\beta_x\delta\beta_y\left(\beta_x^2-\beta_y^2+\beta_z^2\right)}{\lambda_1^2} - \frac{(\gamma-1)B_y\delta\beta_x\beta_y\left(-\beta_x^2+\beta_y^2+\beta_z^2\right)}{\lambda_1^2} - \frac{(\gamma-1)B_z\beta_x\delta\beta_z\left(\beta_x^2+\beta_y^2-\beta_z^2\right)}{\lambda_1^2}\right]$$

$$(F'')^{13} = -\gamma E_x\beta_z + \gamma\beta_x E_z + \frac{1}{\lambda_1^2}\left[B_y\beta_y^2 + \gamma B_y\eta_3^2 - (\gamma-1)B_z\beta_y\beta_z - (\gamma-1)B_x\beta_x\beta_y - \gamma^3 B_x\beta_x^2\delta\beta_x\beta_y - \gamma^3 B_z\beta_y^2\delta\beta_y\beta_z \right.$$
$$-\gamma^3 B_x\beta_x\beta_y^2\delta\beta_y - \gamma^3 B_x\beta_x\beta_y\beta_z\delta\beta_z + \gamma^3\beta_x\beta_z\delta\beta_z E_z + \gamma^3\beta_x^2\delta\beta_x E_z + \gamma\delta\beta_x E_z\eta_2^2 - \gamma\beta_x\beta_y\delta\beta_y E_z - \gamma\beta_x\beta_z\delta\beta_z E_z$$
$$-\gamma^3 E_x\beta_z^2\delta\beta_z - \left(\gamma^2-1\right)\gamma\beta_x\delta\beta_x E_x\beta_z - \left(\gamma^2-1\right)\gamma E_x\beta_y\delta\beta_y\beta_z - \gamma E_x\delta\beta_z\eta_1^2 + \gamma^3\beta_x\beta_y\delta\beta_y E_z - \gamma^3 B_z\beta_y\beta_z^2\delta\beta_z$$
$$+\gamma^3 B_y\beta_x\delta\beta_x\eta_3^2 + \gamma^3 B_y\beta_z\delta\beta_z\eta_3^2 + \gamma^3 B_y\beta_y\delta\beta_y\eta_3^2 - \gamma^3 B_z\beta_x\delta\beta_x\beta_y\beta_z + \frac{2(\gamma-1)B_y\beta_x\delta\beta_x\beta_y^2}{\lambda_1^2} - \frac{2(\gamma-1)B_y\beta_y\delta\beta_y\eta_3^2}{\lambda_1^2}$$
$$+\frac{2(\gamma-1)B_y\beta_y^2\beta_z\delta\beta_z}{\lambda_1^2} + \frac{2(\gamma-1)B_z\beta_x\delta\beta_x\beta_y\beta_z}{\lambda_1^2} - \frac{(\gamma-1)B_z\delta\beta_y\beta_z\left(\beta_x^2-\beta_y^2+\beta_z^2\right)}{\lambda_1^2} - \frac{(\gamma-1)B_z\beta_y\delta\beta_z\left(\beta_x^2+\beta_y^2-\beta_z^2\right)}{\lambda_1^2}$$
$$\left.+\frac{2(\gamma-1)B_x\beta_x\beta_y\beta_z\delta\beta_z}{\lambda_1^2} - \frac{(\gamma-1)B_x\delta\beta_x\beta_y\left(-\beta_x^2+\beta_y^2+\beta_z^2\right)}{\lambda_1^2} - \frac{(\gamma-1)B_x\beta_x\delta\beta_y\left(\beta_x^2-\beta_y^2+\beta_z^2\right)}{\lambda_1^2}\right]$$

$$(F'')^{21} = \gamma E_x\beta_y - \gamma\beta_x E_y + \frac{1}{\lambda_1^2}\left[B_z\beta_z^2 + \gamma B_z\eta_1^2 - (\gamma-1)B_x\beta_x\beta_z - (\gamma-1)B_y\beta_y\beta_z + \gamma^3 B_z\beta_x\delta\beta_x\eta_1^2 - \gamma^3 B_x\beta_x^2\delta\beta_x\beta_z \right.$$
$$-\gamma^3\beta_x^2\delta\beta_x E_y - \gamma^3\beta_x\beta_y\delta\beta_y E_y - \gamma^3\beta_x E_y\beta_z\delta\beta_z + \gamma\beta_x\beta_y\delta\beta_y E_y + \gamma\beta_x E_y\beta_z\delta\beta_z - \gamma\delta\beta_x E_y\eta_2^2 + \gamma E_x\delta\beta_y\eta_3^2$$
$$+\left(\gamma^2-1\right)\gamma E_x\beta_y\beta_z\delta\beta_z + \gamma\left(\gamma^2-1\right)\beta_x\delta\beta_x E_x\beta_y - \gamma^3 B_y\beta_x\delta\beta_x\beta_y\beta_z + \gamma^3 B_z\beta_y\delta\beta_y\eta_1^2 - \gamma^3 B_y\beta_y^2\delta\beta_y\beta_z$$
$$-\gamma^3 B_x\beta_x\beta_y\delta\beta_y\beta_z + \gamma^3 B_z\beta_z\delta\beta_z\eta_1^2 - \gamma^3 B_x\beta_x\beta_z^2\delta\beta_z - \gamma^3 B_y\beta_y\beta_z^2\delta\beta_z + \gamma^3 E_x\beta_y^2\delta\beta_y + \frac{2(\gamma-1)B_z\beta_y\delta\beta_y\beta_z^2}{\lambda_1^2}$$
$$+\frac{2(\gamma-1)B_y\beta_x\delta\beta_x\beta_y\beta_z}{\lambda_1^2} + \frac{2(\gamma-1)B_z\beta_x\delta\beta_x\beta_z^2}{\lambda_1^2} - \frac{(\gamma-1)B_y\delta\beta_y\beta_z\left(\beta_x^2-\beta_y^2+\beta_z^2\right)}{\lambda_1^2} - \frac{2(\gamma-1)B_z\beta_z\delta\beta_z\eta_1^2}{\lambda_1^2}$$
$$-\frac{(\gamma-1)B_x\beta_x\delta\beta_z\left(\beta_x^2+\beta_y^2-\beta_z^2\right)}{\lambda_1^2} - \frac{(\gamma-1)B_x\delta\beta_x\beta_z\left(-\beta_x^2+\beta_y^2+\beta_z^2\right)}{\lambda_1^2} + \frac{2(\gamma-1)B_x\beta_x\beta_y\delta\beta_y\beta_z}{\lambda_1^2}$$
$$\left.-\frac{(\gamma-1)B_y\beta_y\delta\beta_z\left(\beta_x^2+\beta_y^2-\beta_z^2\right)}{\lambda_1^2}\right]$$

3.2 Successively Boosted Frame $\vec{\beta}$ and $\Delta\vec{\beta}$

$$(F''')^{10} = \gamma B_z\beta_y - \gamma B_y\beta_z - \gamma^2\beta_x^2 E_x - \gamma^2\beta_x\beta_z E_z - \gamma^2\beta_x\beta_y E_y + \frac{1}{\lambda_1^2}\left[\gamma^2\beta_x^2 E_x + \gamma E_x\eta_2^2 + \gamma^2\beta_x\beta_y E_y - \gamma\beta_x\beta_y E_y - \gamma^2\beta_x E_z\lambda_5 \right.$$
$$+\gamma^2\beta_x\beta_z E_z - \gamma\beta_x\beta_z E_z + \gamma^2 B_y\beta_x\delta\beta_x\beta_z - \gamma^2 B_z\beta_x\delta\beta_x\beta_y + \gamma B_y\beta_y\delta\beta_y\beta_z - \gamma B_z\beta_y\beta_z\delta\beta_z - (\gamma-1)\gamma B_x\beta_x\delta\beta_y\beta_z$$
$$-\gamma^3 B_y\beta_z\lambda_2 + \gamma B_z\delta\beta_y\left(\gamma\beta_x^2+\beta_z^2\right) - \gamma B_y\delta\beta_z\left(\gamma\beta_x^2+\beta_y^2\right) - \gamma^2\beta_x E_x\lambda_3 - \gamma^3\beta_x\beta_y E_y\lambda_2 + (\gamma-1)\gamma B_x\beta_x\beta_y\delta\beta_z$$
$$\left.-\gamma^2\beta_x E_y\lambda_4 - \gamma^3\beta_x\beta_z E_z\lambda_2 + \gamma^3 E_x\eta_2^2\lambda_2 + \gamma^3 B_z\beta_y\lambda_2\right]$$

$$
\begin{aligned}
(F''')^{20} = {} & \gamma B_x\beta_z - \gamma B_z\beta_x - \gamma^2\beta_x E_x\beta_y - \gamma^2\beta_y^2 E_y - \gamma^2\beta_y\beta_z E_z + \frac{1}{\lambda_1^2}\left[\gamma^3 B_x\beta_y\delta\beta_y\beta_z(\gamma-1)\gamma\beta_x E_x\beta_y + (\gamma-1)\gamma\beta_y\beta_z E_z\right.\\
& +\gamma^3 B_x\beta_x\delta\beta_x\beta_z - \gamma^3 B_z\beta_x^2\delta\beta_x + \gamma^2 B_z\beta_x\beta_y\delta\beta_y + (\gamma-1)\gamma B_y\delta\beta_x\beta_y\beta_z - \gamma B_x\beta_x\delta\beta_x\beta_z + \gamma^2\beta_x\delta\beta_x\beta_y^2 E_y - \gamma^3\beta_y^2\delta\beta_y\beta_z E_z\\
& +\gamma E_y\left(\beta_x^2 + \gamma\beta_y^2 + \beta_z^2\right) - \gamma^3 B_z\beta_x\beta_y\delta\beta_y - \gamma^2 B_x\beta_y\delta\beta_y\beta_z + \gamma B_x\delta\beta_z\left(\beta_x^2 + \gamma\beta_y^2\right) + \gamma B_z\beta_x\beta_z\delta\beta_z - (\gamma-1)\gamma B_y\beta_x\beta_y\delta\beta_z\\
& -\gamma^3 B_z\beta_x\beta_z\delta\beta_z + \gamma^3 B_x\beta_z^2\delta\beta_z - \gamma^3\beta_x^2\delta\beta_x E_x\beta_y - \gamma^3\beta_x E_x\beta_y^2\delta\beta_y + \gamma^2\beta_x E_x\beta_y^2\delta\beta_y - \gamma^2\delta\beta_x E_x\beta_y\eta_2^2 + \gamma^2\beta_x E_x\beta_y\beta_z\delta\beta_z\\
& -\gamma^3\beta_x E_x\beta_y\beta_z\delta\beta_z + \gamma^3\beta_x\delta\beta_x E_y\eta_3^2 - \gamma^2\beta_y\delta\beta_y E_y\eta_3^2 + \gamma^3\beta_y\delta\beta_y E_y\eta_3^2 + \gamma^2\beta_y^2 E_y\beta_z\delta\beta_z + \gamma^3 E_y\beta_z\delta\beta_z\eta_3^2 - \gamma^2\beta_y\delta\beta_z E_z\eta_1^2\\
& \left.-\gamma^3\beta_x\delta\beta_x\beta_y\beta_z E_z - \gamma^3\beta_y\beta_z^2\delta\beta_z E_z + \gamma^2\beta_y^2\delta\beta_y\beta_z E_z + \gamma^2\beta_x\delta\beta_x\beta_y\beta_z E_z - \gamma B_z\delta\beta_x\left(\gamma\beta_y^2 + \beta_z^2\right)\right]
\end{aligned}
$$

$$
\begin{aligned}
(F''')^{30} = {} & \gamma B_y\beta_x - \gamma B_x\beta_y - \gamma^2\beta_x E_x\beta_z - \gamma^2\beta_y E_y\beta_z - \gamma^2\beta_z^2 E_z + \frac{1}{\lambda_1^2}\left[\gamma^3 B_y\beta_x^2\delta\beta_x(\gamma-1)\gamma\beta_x E_x\beta_z + (\gamma-1)\gamma\beta_y E_y\beta_z\right.\\
& +\gamma E_z\left(\beta_x^2 + \beta_y^2 + \gamma\beta_z^2\right) - \gamma^3 B_x\beta_x\delta\beta_x\beta_y + \gamma B_y\delta\beta_x\left(\beta_y^2 + \gamma\beta_z^2\right) + \gamma B_x\beta_x\delta\beta_x\beta_y - (\gamma-1)\gamma B_z\delta\beta_x\beta_y\beta_z - \gamma B_y\beta_x\beta_y\delta\beta_y\\
& +\gamma^3 B_y\beta_x\beta_y\delta\beta_y - \gamma^2 B_y\beta_x\beta_z\delta\beta_z + (\gamma-1)\gamma B_z\beta_x\delta\beta_y\beta_z - \gamma B_x\delta\beta_y\left(\beta_x^2 + \gamma\beta_z^2\right) - \gamma^3 B_x\beta_y\beta_z\delta\beta_z + \gamma^3 B_y\beta_x\beta_z\delta\beta_z\\
& +\gamma^2 B_x\beta_y\beta_z\delta\beta_z - \gamma^3\beta_x^2\delta\beta_x E_x\beta_z - \gamma^3\beta_x E_x\beta_y\delta\beta_y\beta_z + \gamma^2\beta_x E_x\beta_y\delta\beta_y\beta_z - \gamma^2\delta\beta_x E_x\beta_z\eta_2^2 + \gamma^2\beta_x E_x\beta_z^2\delta\beta_z\\
& -\gamma^3\beta_x E_x\beta_z^2\delta\beta_z - \gamma^3\beta_x\delta\beta_x\beta_y E_y\beta_z - 2\gamma^3\beta_y^2\delta\beta_y E_y\beta_z - 2\gamma^2\delta\beta_y E_y\beta_z\eta_3^2 + \gamma^2\beta_x\delta\beta_x\beta_y E_y\beta_z + \gamma^2\beta_y E_y\beta_z^2\delta\beta_z\\
& -\gamma^3\beta_y E_y\beta_z^2\delta\beta_z + \gamma^3\beta_y\delta\beta_y E_z\eta_1^2 + \gamma^3\beta_z\delta\beta_z E_z\eta_1^2 + \gamma^3\beta_x\delta\beta_x E_z\eta_1^2 + \gamma^2\beta_x\delta\beta_x\beta_z^2 E_z - \gamma^2\beta_z\delta\beta_z E_z\eta_1^2 - \gamma^3 B_x\beta_y^2\delta\beta_y\\
& \left.+\gamma^2\beta_y\delta\beta_y\beta_z^2 E_z\right]
\end{aligned}
$$

$$
\begin{aligned}
(F''')^{32} = {} & \gamma E_y\beta_z - \gamma\beta_y E_z + \gamma^3\beta_y\delta\beta_y E_y\beta_z - \gamma^3\beta_y^2 E_y\delta\beta_z + \gamma^3\delta\beta_y\beta_z^2 E_z - \gamma^3\beta_y\beta_z\delta\beta_z E_z + \frac{1}{\lambda_1^2}\left[-\gamma^2 B_y\beta_x\lambda_4 + \gamma^3 B_x\eta_2^2\lambda_2\right.\\
& -(\gamma-1)B_z\beta_x\beta_z + B_x\left(\beta_x^2 + \gamma\left(\beta_y^2 + \beta_z^2\right)\right) + (\gamma-1)\gamma\beta_x E_x\delta\beta_y\beta_z - (\gamma-1)\gamma\beta_x E_x\beta_y\delta\beta_z - \gamma^2 B_z\beta_x\lambda_5 - \gamma^3 B_z\beta_x\beta_z\lambda_2\\
& +\gamma^2 E_y\delta\beta_z\left(\beta_x^2 + \gamma\left(\beta_y^2 + \beta_z^2\right)\right) - \gamma^2\delta\beta_y E_z\left(\beta_x^2 + \gamma\left(\beta_y^2 + \beta_z^2\right)\right) + (\gamma-1)\gamma^2\beta_x\delta\beta_x E_y\beta_z - (\gamma-1)B_y\beta_x\beta_y - \gamma^3 B_y\beta_x\beta_y\lambda_2\\
& \left.-\gamma^2 B_x\beta_x\lambda_3 - (\gamma-1)\gamma^2\beta_x\delta\beta_x\beta_y E_z\right]
\end{aligned}
$$

$$
\begin{aligned}
(F''')^{13} = {} & \gamma\beta_x E_z - \gamma E_x\beta_z - \gamma^3\beta_x\delta\beta_x E_x\beta_z + \gamma^3\beta_x^2 E_x\delta\beta_z + \gamma^3\beta_x\beta_z\delta\beta_z E_z + \frac{1}{\lambda_1^2}\left[\gamma^3 B_y\eta_3^2\lambda_2 - \gamma^2 B_x\beta_y\lambda_3 - \gamma^3 B_x\beta_x\beta_y\lambda_2\right.\\
& +\gamma\delta\beta_x\beta_z^2 E_z - (\gamma-1)B_x\beta_x\beta_y - (\gamma-1)B_z\beta_y\beta_z - \gamma^2 B_y\beta_y\lambda_4 - \gamma^2\delta\beta_x\beta_y E_y\beta_z - \gamma^2 B_z\beta_y\lambda_5 - \gamma\beta_x\beta_y E_y\delta\beta_z\\
& -\gamma^3 B_z\beta_y\beta_z\lambda_2 - \gamma^2 E_x\beta_y^2\delta\beta_z - (\gamma-1)\gamma^2 E_x\beta_y\delta\beta_y\beta_z - \gamma^3 E_x\delta\beta_z\eta_3^2 + \gamma^2\beta_x\beta_y E_y\delta\beta_z + \gamma^3\beta_x^2\delta\beta_x E_z + \gamma^2\delta\beta_x\beta_y^2 E_z\\
& \left.+\gamma\delta\beta_x\beta_y E_y\beta_z + (\gamma-1)\gamma^2\beta_x\beta_y\delta\beta_y E_z + B_y\left(\gamma\beta_x^2 + \beta_y^2 + \gamma\beta_z^2\right)\right]
\end{aligned}
$$

$$
\begin{aligned}
(F''')^{21} = {} & \gamma^3\beta_x\delta\beta_x E_x\beta_y - \gamma^3\beta_x^2 E_x\delta\beta_y + \gamma E_x\beta_y + \gamma^3\delta\beta_x\beta_y^2 E_y - \gamma\beta_x E_y - \gamma^3\beta_x\beta_y\delta\beta_y E_y + \gamma^3\delta\beta_x\beta_y\beta_z E_z - \gamma^3\beta_x\delta\beta_y\beta_z E_z\\
& + \frac{1}{\lambda_1^2}\left[\gamma^3 B_z\eta_1^2\lambda_2 - (\gamma-1)B_x\beta_x\beta_z - (\gamma-1)B_y\beta_y\beta_z + B_z\left(\gamma\beta_x^2 + \gamma\beta_y^2 + \beta_z^2\right) - \gamma^3 B_x\beta_x\beta_z\lambda_2 - \gamma^3 B_y\beta_y\beta_z\lambda_2\right.\\
& +\gamma^3 E_x\delta\beta_y\eta_1^2 - \gamma^3\delta\beta_x E_y\eta_1^2 - \gamma^2 B_x\beta_z\lambda_3 - \gamma^2 B_y\beta_z\lambda_4 - \gamma^2 B_z\beta_z\lambda_5 + (\gamma-1)\gamma^2 E_x\beta_y\beta_z\delta\beta_z - (\gamma-1)\gamma^2\beta_x E_y\beta_z\delta\beta_z\\
& \left.+(\gamma-1)\gamma^2\beta_z E_z\lambda_6 + \gamma^2 E_x\delta\beta_y\beta_z^2 - \gamma^2\delta\beta_x E_y\beta_z^2\right]
\end{aligned}
$$

4 Comparison with the References

As mentioned in the Introduction, this work is inspired by Ungar et al.[9–11,13–16] so it is natural to compare our approach with Ungar to see if we share the same overall features. To check that we will work for a 2-dimensional case for simplicity by letting $\beta_y = \beta_z = \delta\beta_z = 0$ in the first part of Eq. (38) of the main article which will give[6]:

$$
A(\Delta\vec{\beta}) = \begin{pmatrix} 1 & -\gamma^2\delta\beta_x & -\gamma\delta\beta_y & 0 \\ -\gamma^2\delta\beta_x & 1 & 0 & 0 \\ -\gamma\delta\beta_y & 0 & 1 & 0 \\ 0 & 0 & 0 & 1 \end{pmatrix} \tag{2}
$$

From $A(\Delta\vec{\beta})$, the relativistic velocity composition can be easily extracted:

$$\Delta\vec{\beta} = \gamma^2 \delta\beta_x \hat{x} + \gamma\delta\beta_y \hat{y} \tag{3}$$

Now for the three different inertial frames discussed in the Introduction Σ, Σ' and Σ'', we have

Relative velocity of Σ' with respect to $\Sigma : \vec{\beta}$
Relative velocity of Σ'' with respect to $\Sigma' : \Delta\vec{\beta}$

We can now use the relativistic velocity composition rule mentioned in Eq. (8) of the main article to calculate the relative velocity of Σ'' with respect to Σ[9–11,13–16] by defining:

$$\begin{aligned} \vec{u} &= \vec{\beta} = \beta\hat{x} \\ \vec{v} &= \Delta\vec{\beta} = \gamma^2\delta\beta_x\hat{x} + \gamma\delta\beta_y\hat{y} \\ \gamma_u &= \gamma \\ \gamma_v &\approx 1 \end{aligned}$$

After plugging in the values and simplifying, we get:

$$\begin{aligned} (\vec{u}\oplus\vec{v})_x &= \frac{\beta + \gamma^2\delta\beta_x}{1+\gamma^2\beta\delta\beta_x} \\ (\vec{u}\oplus\vec{v})_y &= \frac{\delta\beta_y}{1+\gamma^2\beta\delta\beta_x} \\ \gamma_{\vec{u}\oplus\vec{v}} &\approx \gamma(1+\gamma^2\beta\delta\beta_x) \end{aligned} \tag{4}$$

Constructing the boost matrix from the above equation:

$$A(\vec{\beta}+\delta\vec{\beta}) = \begin{pmatrix} \gamma+\gamma^3\beta\delta\beta_x & -(\gamma\beta+\gamma^3\delta\beta_x) & -\gamma\delta\beta_y & 0 \\ -(\gamma\beta+\gamma^3\delta\beta_x) & \gamma+\gamma^3\beta\delta\beta_x & (\gamma-1)\frac{\delta\beta_y}{\beta} & 0 \\ -\gamma\delta\beta_y & (\gamma-1)\frac{\delta\beta_y}{\beta} & 1 & 0 \\ 0 & 0 & 0 & 1 \end{pmatrix} \tag{5}$$

We can compare this boost matrix with Eq. (40) of the main article and notice that they are completely identical. Therefore our approach gives the identical final result as discussed in[9–11,13–16].

References

1. Thomas, L. H. The Motion of the Spinning Electron. *Nature*, **117,** 514 (1926); *Phil. Mag.* **3**, 1 (1927).

2. Jackson, J. D. *Classical Electrodynamics*, 524-527 (John Wiley & Sons, Inc., 1999).

3. Jackson, J. D. *Classical Electrodynamics*, 530-532 (John Wiley & Sons, Inc., 1999).

4. Jackson, J. D. *Classical Electrodynamics*, 539-540 (John Wiley & Sons, Inc., 1999).

5. Jackson, J. D. *Classical Electrodynamics*, 546-548 (John Wiley & Sons, Inc., 1999).

6. Jackson, J. D. *Classical Electrodynamics*, 550-552 (John Wiley & Sons, Inc., 1999).

7. Jackson, J. D. *Classical Electrodynamics*, 556-557 (John Wiley & Sons, Inc., 1999).

8. Jackson, J. D. *Classical Electrodynamics*, 558-559 (John Wiley & Sons, Inc., 1999).

9. Ungar, A. A. Thomas rotation and the parametrization of the Lorentz transformation group. *Found. Phys. Lett.* **1**, 57-89, https://doi.org/10.1007/BF00661317 (1988).

10. Ungar, A. A. The relativistic velocity composition paradox and the Thomas rotation. *Found. Phys.* **19**, 1385-1396, https://doi.org/10.1007/BF00732759 (1989).

11. Ungar, A. A. The Relativistic Noncommutative Nonassociative Group of Velocities and the Thomas Rotation. *Results. Math.* **16**, 168-179, https://doi.org/10.1007/BF03322653 (1989).

12. Ungar, A. A. Axiomatic approach to the nonassociative group of relativistic velocities. *Found. Phys. Lett.* **2**, 199-203, https://doi.org/10.1007/BF00696113 (1989).

13. Ungar, A. A. Successive Lorentz transformations of the electromagnetic field. *Found. Phys.* **21**, 569-589, https://doi.org/10.1007/BF00733259 (1991).

14. Ungar, A. A. Thomas precession and its associated grouplike structure. *Am. J. Phys.* **59**, 824, https://doi.org/10.1119/1.16730 (1991).

15. Ungar, A. A. Thomas precession: Its underlying gyrogroup axioms and their use in hyperbolic geometry and relativistic physics. *Found. Phys.* **27**, 881-951, https://doi.org/10.1007/BF02550347 (1997).

16. Ungar, A. A. The Relativistic Composite-Velocity Reciprocity Principle. *Found. Phys.* **30**, 331-342, https://doi.org/10.1023/A:1003653302643 (2000).

17. Lamoreaux, S. K. & Golub, R. Detailed discussion of a linear electric field frequency shift induced in confined gases by a magnetic field gradient: Implications for neutron electric-dipole-moment experiments. *Phys. Rev. A*, **71,** 032104 (2005).

18. Barabanov, A. L. Golub, R. & Lamoreaux, S. K. Electric dipole moment searches: Effect of linear electric field frequency shifts induced in confined gases. *Phys. Rev. A*, **74,** 052115 (2006).

19. Pignol, G. Guigue, M. Petukhov, A. & Golub, R. Frequency shifts and relaxation rates for spin-1/2 particles moving in electromagnetic fields. *Phys. Rev. A*, **92,** 053407 (2015).
